# Acute gastrointestinal permeability after traumatic brain injury in mice precedes a bloom in *Akkermansia muciniphila* supported by intestinal hypoxia

**DOI:** 10.1038/s41598-024-53430-4

**Published:** 2024-02-05

**Authors:** Anthony J. DeSana, Steven Estus, Terrence A. Barrett, Kathryn E. Saatman

**Affiliations:** 1 Department of Physiology, University of Kentucky, Biomedical and Biological Sciences Research Building (BBSRB), B473, 741 South Limestone St., Lexington, KY 40536 USA; 2 Spinal Cord and Brain Injury Research Center, University of Kentucky, Biomedical and Biological Sciences Research Building (BBSRB), B473, 741 South Limestone St., Lexington, KY 40536 USA; 3 Division of Digestive Diseases and Nutrition, Department of Internal Medicine – Digestive Health, University of Kentucky, Lexington, KY 40536 USA; 4 Department of Microbiology, Immunology and Molecular Genetics, University of Kentucky, Medical Science Building, MN649, 780 Rose St., Lexington, KY 40536 USA; 5 Sanders Brown Center on Aging, University of Kentucky, Lee T. Todd, Jr. Building, Rm: 537, 789 South Limestone St., Lexington, KY 40536 USA

**Keywords:** DNA sequencing, Animal disease models, Bioinformatics, Mouse, Fluorescence imaging, Optical imaging, Neurodegeneration, Microbial communities, Microbiota, Large intestine, Colon, Small intestine, Ileum, Experimental models of disease

## Abstract

Traumatic brain injury (TBI) increases gastrointestinal morbidity and associated mortality. Clinical and preclinical studies implicate gut dysbiosis as a consequence of TBI and an amplifier of brain damage. However, little is known about the association of gut dysbiosis with structural and functional changes of the gastrointestinal tract after an isolated TBI. To assess gastrointestinal dysfunction, mice received a controlled cortical impact or sham brain injury and intestinal permeability was assessed at 4 h, 8 h, 1 d, and 3 d after injury by oral administration of 4 kDa FITC Dextran prior to euthanasia. Quantification of serum fluorescence revealed an acute, short-lived increase in permeability 4 h after TBI. Despite transient intestinal dysfunction, no overt morphological changes were evident in the ileum or colon across timepoints from 4 h to 4 wks post-injury. To elucidate the timeline of microbiome changes after TBI, 16 s gene sequencing was performed on DNA extracted from fecal samples collected prior to and over the first month after TBI. Differential abundance analysis revealed that the phylum *Verrucomicrobiota* was increased at 1, 2, and 3 d after TBI. The *Verrucomicrobiota* species was identified by qPCR as *Akkermansia muciniphila*, an obligate anaerobe that resides in the intestinal mucus bilayer and produces short chain fatty acids (e.g. butyrate) utilized by intestinal epithelial cells. We postulated that TBI promotes intestinal changes favorable for the bloom of *A. muciniphila*. Consistent with this premise, the relative area of mucus-producing goblet cells in the medial colon was significantly increased at 1 d after injury, while colon hypoxia was significantly increased at 3 d. Our findings reveal acute gastrointestinal functional changes coupled with an increase of beneficial bacteria suggesting a potential compensatory response to systemic stress after TBI.

## Introduction

Traumatic brain injury (TBI) is a major source of death and disability with approximately 2.8 million people suffering a TBI annually in the United States alone^1,2^. The initial brain insult and ensuing secondary injury cascade result in complex damage and dysfunction not only to the brain but also to other organ systems^3,4^. Increasing evidence suggests that the neuroenteric axis is altered following TBI, compromising bidirectional coordination of external and internal stimuli by the central and enteric nervous systems^5,6^. Gastrointestinal (GI) dysfunction, including dysmotility, fecal incontinence and sepsis, is well documented in the TBI population^7–9^. Indeed, at one or more years after injury, TBI is associated with a 2.5-fold increase in the incidence of digestive system conditions and 12-fold increase in death due to septicemia^9,10^. Rodent models of TBI recapitulate aspects of GI dysfunction seen in humans, including altered transit time^11^ and increased intestinal permeability^5,12^. Such perturbations to normal GI function may result in disrupted nutrient absorption and bacterial translocation, potentially contributing to deleterious central nervous system outcomes^5,6,13,14^.

The microbiota residing in the intestinal tract mediate signaling along the neuroenteric axis^15^. The cells of the small and large intestine interact with microbes and microbial metabolites to affect direct and indirect signaling to the central nervous system. The gut’s native bacterial populations are critical for maintaining GI health by contributing to intestinal metabolism and digestion and influence many systems critical for normal development of the brain and immune system^16–19^. Changes to the gut microbiome have been reported after TBI, although the specific findings vary (reviewed in^20^). Experimental perturbation of the microbiome worsens outcomes after experimental TBI^6,21,22^. More specifically, TBI-associated pathology and neurologic deficits are exacerbated by the induction of colitis early after injury^22^ and with the introduction of intestinal pathogens at chronic timepoints^6^. Antibiotic-induced dysbiosis after experimental TBI is associated with greater neuroinflammation and reduced hippocampal neurogenesis, compromising potentially reparative neuroplasticity^21^. Given that gut-to-brain signaling can affect multiple outcomes, it is important to understand the brain-to-gut changes that are initiated after TBI.

TBI can affect not only GI function and the gut microbiome, but also the structural integrity of the GI tract. Acute small intestinal villi blunting and chronic thickening of the muscularis mucosae have been noted after experimental brain injury^5,6,13^. Moreover, due to the close association between the gut microbiota and the intestinal epithelium, deleterious changes to the gut microbiome are often associated with damage to the intestinal tract and vice versa^23–25^.

The intestinal microbiome has been posited as a potential therapeutic target after TBI^26^, but how the GI tract is altered by TBI is still poorly understood. To test the hypothesis that TBI initiates intestinal dysfunction and damage in concert with gut dysbiosis, we induced a controlled cortical impact (CCI) brain injury in mice. Damage to the GI tract was assessed at multiple timepoints over a 4 wk post-injury period by quantifying morphological features of crypt-villi and crypt structures and goblet cells in the small and large intestines. The fecal microbiome was then profiled at corresponding timepoints by performing 16 s rRNA gene sequencing.

## Methodology

### Subjects

C57BL/6J male mice were obtained from Jackson Laboratories at 4–8 wks of age (n_total_ = 125). Mice were allowed to acclimate within the University of Kentucky Medical Center animal vivarium for at least one week prior to any procedures. The vivarium was maintained at a constant temperature with a 14/10 h light/dark cycle. Unless otherwise stated, mice were provided food and water ad libitum.

For microbiome studies, mice were co-housed by injury condition following inoculation with a common microbiome to minimize inter-individual variation (see pre-injury microbiome normalization methods for additional information). All other mice were co-housed regardless of injury condition with their cage-mates as determined upon arrival to the vivarium.

All procedures were approved by the University of Kentucky’s Institutional Animal Care and Use Committee, under IACUC protocol 2019-3170, and all experiments were performed in accordance with the “Guide for the Care and Use of Laboratory Animals” and relevant regulations^27^.

### CCI surgery, group randomization

Adult mice (8–10 wks of age) were randomized into CCI or sham injury groups using a random list generator (random.org). CCI mice received a moderate brain injury (1.1 mm depth, 3.5 m/s speed) as previously described^28^. In short, mice were initially anesthetized using 3% isoflurane and placed in a stereotaxic frame (David Kopf Instruments). Once the head was stabilized, anesthesia was maintained at 2.5% isoflurane for the duration of the surgery. The scalp was shaved and disinfected prior to analgesic administration via subcutaneous injection (0.5% bupivacaine with 1:200,000 epinephrine in sterile saline; 2 mg/kg bodyweight). A midline incision was made and the scalp retracted. A 5 mm diameter craniotomy was made over the left parietal cortex midway between bregma and lambda. The 3 mm diameter, rounded impactor tip was centered over the craniotomy and a cortical contusion was produced using an electromagnetically driven impactor device (Leica Biosystems). The craniotomy site was then closed with a cranioplasty to protect the brain from further damage. Following surgery, mice were placed on a heated pad to maintain body temperature until they were ambulatory. Sham-injured mice served as the control group and underwent all procedures except the impact. A priori power analysis was not performed because GI histology outcomes had not been assessed previously within the lab. Thus, group sizes were based upon published data from TBI studies assessing GI tissue outcomes^5,6,29^. Group sizes for each experimental parameter are provided in Supplemental Table 1.

### Intestinal permeability assay

Intestinal permeability was assessed as previously published^30^ in mice that were euthanized at 4 h, 8 h, 1 d, or 3 d after sham injury or CCI (n_sham_ = 2–3/timepoint; n_CCI_ = 6–9/timepoint). All mice were fasted approximately 10–13 h prior to euthanasia to allow for complete clearance of the GI tract. Mice designated for the 4 h group were fasted overnight prior to surgery. Mice surviving 8 h were fasted beginning 2 h prior to surgery. To assess permeability, 4 kDa FITC dextran (100 mg/ml; FD4, Sigma Aldrich, CAS: 60842-46-8) was administered 4 h prior to euthanasia by oral gavage at a dose of 44 mg/100 g body weight. Mice designated for euthanasia 4 h after injury were gavaged directly after they regained consciousness from surgery. A single sham mouse from the 4 h assessment group was excluded due to a gavaging error.

Mice were euthanized by overdose of sodium pentobarbital (Fatal Plus, 150 mg/kg i.p.). A midline laparotomy was then immediately performed, followed by a thoracotomy to expose the heart for blood collection from the left atria. If insufficient blood was collected, the right auricle was clipped and pooled blood was collected. Mice were excluded if an insufficient amount of blood was able to be collected after clipping the right auricle (Sham: n_8hr_ = 1, n_1d_ = 1, n_3d_ = 1 | CCI: n_1d_ = 1, n_3d_ = 2). Blood was allowed to clot at room temperature and was then spun down in a centrifuge (4 °C; 1500xG for 15–20 min). Serum was stored at -20 °C until analysis. After blood collection, mice were perfused with heparinized saline and tissue collection proceeded as outlined below. Serum fluorescence was measured on a plate reader (Synergy | HTX multi-mode reader; Gen5 Image+) and concentration of FITC dextran was determined based upon a standard curve.

### Pimonidazole-HCl administration

To assess intestinal hypoxia after TBI (n_sham_ = 11, n_CCI_ = 6–7/timepoint), mice received an intraperitoneal (i.p.) injection of 60 mg/kg pimonidazole-HCl (Hypoxyprobe-1/HP1 | Hypoxyprobe, Inc.) 1 h prior to euthanasia as per manufacturer instructions. Under hypoxic conditions, HP1 is reduced and forms protein adducts that can then be detected using antibodies against this protein adduct^31^.

### Euthanasia, tissue collection and processing

Animals were euthanized with an overdose of sodium pentobarbital (Fatal Plus, 150 mg/kg i.p.), and were transcardially perfused with heparinized saline. The small and large intestine were dissected out together for processing (described below) before resuming perfusion with 10% buffered formalin. As previously described^28^, brains were removed from the skull after an overnight post-fix in 10% buffered formalin, and further post-fixed for 24 h prior to cryoprotection with 30% sucrose solution. Brains were snap frozen and stored at -80 °C. Brains were cut coronally at 40 μm thickness using a freezing, sliding microtome (Microm HM430; Thermo Scientific) and stained with Cresyl Echt Violet to assess gross histology. After evaluation by two blinded investigators without knowledge of other outcome parameters, one sham mouse was excluded from the study due to substantial damage to the brain along the perimeter of the craniotomy. One mouse euthanized 4 h after CCI was excluded due to an absence of cortical tissue damage, implicating technical failure of the impact.

Following removal of the intact small intestine and colon, the ileum was identified as the distal third of the small intestine and separated. For the colon, the cecum was excluded and the medial and distal portions of the colon were dissected out together for further processing. The dissected ileum and colon were each Swiss rolled onto syringe plungers^32^ before overnight post-fixation in 10% buffered formalin. Swiss-rolled tissue was then pinned and transferred to 70% ethanol for paraffin embedding. Tissue was cut on a rotary microtome (Finesse; Shandon) at a 4 μm thickness and mounted on positively charged slides. Tissue was air dried overnight and then baked overnight at 50ºC.

### Histology, immunohistochemistry and image analysis

Histology was performed on GI tissue collected from animals generated for several experiments within the present study (n_sham_ = 3–6/timepoint; n_CCI_ = 6–10/timepoint). Animals euthanized at 4 h, 8 h, 1 d, and 3 d after injury were also utilized for the FITC dextran permeability assay described above, while the 1 wk and 2 wk mice were generated for the specific purpose of GI tissue analysis. Tissue for the 4 wk timepoint was collected from the animals that were utilized for the microbiome studies. Analysis of HP1 immunofluorescence was performed on the 1d and 3d groups. All tissue processing, staining, immunolabeling, and measurements were taken by a researcher blinded to brain injury status and timepoint.

#### Alcian Blue staining

Swiss-rolled intestinal tissue sections were stained with Alcian Blue (VECTOR labs | H-3501), as per manufacturer instructions, to label acidic mucins with a pH < 2.5. Tissue was then counterstained with nuclear fast red. Slides were scanned at 20 × with a Zeiss Axio Scan Z.1 digital slide scanner allowing for a single high-resolution, zoomable image of each slide.

#### Quantification of intestinal morphological parameters

Intestinal tissue stained with Alcian Blue and nuclear fast red was used to assess alterations to the morphology of both the small and large intestine. The functional unit within the ileum is the crypt-villi structure, which consists of a villus surrounded by invaginations known as crypts. Within the colon, the medial region (ascending colon) was differentiated from the distal region (descending colon) based on the presence of transverse folds. As crypt structures within the medial colon are shorter than those of distal colon, crypt depth was measured separately for these regions. Either increased or decreased ileum crypt-villi distance or colon crypt depth is indicative of damage to the GI tract and can be associated with altered nutrient absorption, damage and inflammation^33–36^.

Within the HALO image analysis platform (version 3.2; Indica Labs), all well-oriented ileum crypt-villi and colon crypt structures, as determined using published criteria^37^, were measured along the length of the Swiss roll. The length from the tip of the villus to the base of that villi’s crypts was measured in the ileum while the distance from the luminal edge of the crypt to its base was measured in the colon. The minimum number of crypt-villi structures quantified per ileum was 20. Then minimum number of crypts measured per colon region was 10. Crypt-villi distance and crypt depth values were averaged for each animal.

#### Alcian Blue staining quantification

For the ileum, Alcian Blue-labeled goblet cells were manually counted within well-oriented crypt-villi structures within five randomly placed square fields (area = 1.5 mm^2^). Images of well-oriented villi within the field were exported at a resolution of 5000 pixels/in using the “Figure Maker” tool in HALO for manual counting within Adobe Photoshop (version 4 for iPad; Adobe). Goblet cells were counted if they had positive staining and a clearly identifiable nucleus.

Within the colon, individual well-oriented crypts within the medial and distal colon were manually outlined using HALO software. Due to the tight packing of goblet cells, colonic goblet cells were quantified using the Area Quantification v1.0 algorithm within HALO to determine the area of Alcian Blue-positive tissue relative to the individual crypt area. This algorithm was adjusted per staining run to account for lighter or darker Alcian Blue labeling. The percent Alcian Blue-positive staining was averaged across crypts separately within the medial and the distal colon per animal.

#### Colon hypoxia immunohistochemistry

Swiss-rolled intestinal tissues were deparaffinized using xylenes and rehydrated through an ethanol gradient into ddH_2_O. To perform antigen retrieval, slides were placed in a 1 × citric acid antigen unmasking solution (pH 6.0; VECTOR Labs | H-3300) for 4 min in a pressure cooker (Instant Pot LUX; Instant Pot Company). After pressure was released, slides were cooled to room temperature before washing in 1 × TBS. Tissue was blocked in 5% normal horse serum (NHS) in 0.1% Triton X-100 in 1 × TBS (TBS-T) before overnight incubation in biotinylated mouse anti-HP1 mAb (1:50 | Hypoxyprobe, Inc) or rabbit anti-HIF1α pAb (1:100 | Abcam) within a humidified chamber at 4 °C or at room temperature. After washing in 1 × TBS, tissue labeled with anti-HIF1α was incubated in donkey anti-rabbit biotin (1:1000 | Jackson ImmunoResearch). Slides labeled against HP1 or HIF1α were then incubated in streptavidin Alexa 594 (1:1000 | Invitrogen). Omission of primary antibody served as a negative control. HP1 labeled slides were scanned on a Zeiss Axio Scan Z.1 digital slide scanner at 20x while HIF1α labeled slides were imaged on a Nikon C2plus confocal microscope at 10x. HP1 and HIF1α provide similar labeling patterns in the colon^38^.

#### Colon hypoxia quantification

Healthy colon tissue is more hypoxic at the luminal edge compared to the crypt base^39^. To determine the effects of CCI on this hypoxic gradient, three visual fields with at least three well-oriented crypts were identified within the distal colon and exported from HALO using the “Figure Maker” tool. These images were imported into FIJI (ImageJ version 1.53 k^40^) to plot the pixel intensity profile from luminal edge to crypt base as a means of assessing both the degree (intensity) and depth of hypoxia. Using the line tool, two lines were drawn at the edges of each crypt from the luminal edge to the base of the crypt. After all lines were drawn, a gaussian blur was applied (σ radius = 3 μm) to minimize the effect that unlabeled goblet cells had on the pixel intensity profile. The pixel intensity gradient was plotted for the red channel using the “plot profile” tool. Because each intensity profile consisted of differing numbers of X, Y coordinates, points were interpolated within R to provide Y-values along an X-axis normalized to a percent distance (0–100% with 1% increments). The two interpolated hypoxia profiles for each crypt were then averaged to provide an overall pixel intensity profile of hypoxia from luminal edge to crypt base. Crypt hypoxia profiles were averaged within a field. Field hypoxia profiles were then averaged for each animal. Hypoxia intensity and depth profiles were imported from R to GraphPad Prism 9 (GraphPad Software) to calculate the area under the curve and peak intensity after subtracting the baseline of each curve.

### Pre-injury microbiome normalization and fecal collection

Because preliminary work suggested pre-injury microbiome variation between cages (data not shown), we normalized the microbiome of all mice prior to injury by flushing the GI tract with the osmotic laxative polyethylene glycol (PEG; Macrogol 4000, 425 g/L | Fortrans) as per the paradigm described by Wrzosek et al.^41^. The mice were then inoculated with cecal contents from age and sex matched mice as described below. Only mice utilized as part of microbiome experiments underwent microbiome normalization.

#### Cecal content collection

Conventionally raised, male mice were used as cecal content donors (n = 10). To allow for stabilization of their microbiome within our facilities, mice were acclimated to the vivarium with normal bedding and cage changes for 3 wks after their arrival. Following euthanasia at 8–9 wks of age, the cecum was rapidly removed and its contents extruded into a sterile, DNase-free, polypropylene tube. Equal parts, by weight, from each animal were mixed and vortexed to a homogenate that was aliquoted and stored at − 80 °C until reconstitution for administration.

#### Whole bowel irrigation and microbiome transplant

**Figure 1:**
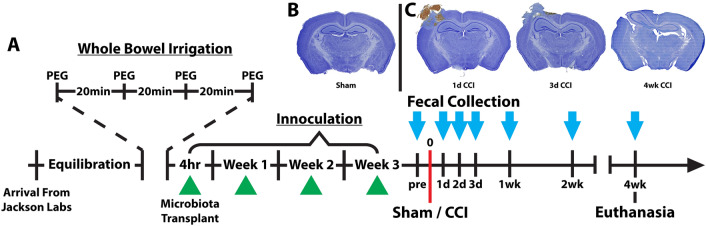
Study timeline of microbiome normalization, TBI and fecal collection. (**A**) Prior to fecal collection, the microbiome was normalized by performing a whole bowel irrigation with polyethylene glycol (PEG) followed by repeated inoculations with a cecal slurry (green triangles) collected from age and sex-matched mice. Fecal samples were collected (light blue arrow) prior to injury and at several timepoints out to 4 wks post-injury. Examples of Nissl stain from (**B**) a sham control and (**C**) controlled cortical impact (CCI) mice at early and chronic timepoints illustrate injury progression from a hemorrhagic contusion to cortical cavitation.

Five-week-old mice were allowed to acclimate for one week prior to the three-week whole bowel irrigation procedure. This allowed for mice to be injured within our target age range of 8–10 wks of age. One hour prior to whole bowel irrigation, mice were placed in a clean, bedding-free cage to prevent coprophagy, with free access to water. Mice were gavaged four times at 20 min intervals with 200 µl of PEG solution^41^. This procedure visibly cleared the contents from the large intestine and was effective in reducing overall DNA content within the colon’s luminal contents (Supplemental Fig. 1). Cecal content homogenates were reconstituted immediately prior to gavage by thawing the homogenates in a warm water bath and rapidly diluting 100-fold in sterile 1 × PBS. Animals were gavaged with reconstituted cecal material 4 h after the final PEG gavage, and again weekly for the next 3 wks (Fig. 1A). Throughout the inoculation period, additional steps were taken to minimize microbiome differences prior to injury by using a mixed bedding approach^42^. Prior to injury, all animals were co-housed, and at cage change, soiled bedding from all cages were mixed and redistributed in equal parts with fresh bedding. The week after the final microbiota inoculation, animals underwent sham or CCI injury as described above. Following surgery, mice were co-housed by injury group and the mixed bedding approach was continued within each injury group.

Weight of animals was monitored with each inoculation, 1 and 2 d prior to injury, on the date of surgery, and then again at each fecal collection timepoint after injury. Body weight changes across time were equivalent for mice randomized into sham and CCI groups (Supplemental Fig. 2).

### Microbiome analyses

#### Read processing and alpha and beta diversity

Adapter sequences were trimmed within the Illumina MiSeq platform and the resultant raw reads were imported into R (version 3.6.3-4.1.2 as released). The raw and processed 16s rRNA gene sequencing data discussed within this document have been deposited in the NCBI Gene Expression Omnibus^43^ and are accessible through the accession number GSE239472 (https://www.ncbi.nlm.nih.gov/geo/query/acc.cgi?acc=GSE239472). All pre-processing and processing was conducted in R using RStudio^44^, based on the workflow outlined by Callahan et al.^45^. Low quality sequences were filtered out and the remaining sequences were trimmed to a consistent length by trimming the first 13 bases and truncating the final 10 bases as determined by read quality profiles. Forward and reverse reads were joined and sequence variants inferred using Divisive Amplicon Denoising Algorithm (DADA2) to generate an amplicon sequence variant (ASV) table^46^. Chimeric sequences were removed and samples containing fewer than 10,000 high quality reads were excluded from further downstream analysis. Two samples from a single sham animal (3 d and 2 wk) were excluded for having low read counts prior to filtering. Prior to filtering there was an average of 137,413 reads per sample (minimum = 23,253 | maximum = 320,550) with an average of 29% of reads being discarded as low quality. After filtering for low-quality reads there was average of 98,351 reads per sample with a minimum number of reads of 11,691 and a maximum of 320,550. Taxonomy was assigned using the SILVA reference database (v132) and a phylogenetic tree was constructed using DECIPHER^47^. Within the Phyloseq package^48^, the ASV table, sample metadata, taxonomy information, and phylogenetic tree were merged into a Phyloseq object for diversity analysis.

Diversity metrics were calculated in R primarily utilizing the Phyloseq and Microbiome packages^48,49^. Due to the high level of variability in read depth, alpha diversity metrics were calculated using data rarefied to a read depth of 10,000. To assess beta diversity, ASVs were transformed into relative abundance values and “distance” values were generated for three commonly used beta diversity metrics: Bray Curtis dissimilarity, UniFrac distance, and Weighted UniFrac distance. Diversity metrics have been reviewed by Kers and Saccenti^50^.

#### Differential abundance analysis (ANCOM-BC)

Differential abundance analysis was conducted using Analysis of Compositions of Microbiomes with Bias Correction (ANCOM-BC v1.2) within R^51^. Importantly, this analysis accounts for the compositionality that is inherent to microbiome data sets^52^. Additionally, this analysis controls for the false discovery rate (FDR) and reports adjusted p-values (q-values). This analysis was conducted at different taxonomic levels by collapsing the ASV table by taxonomic rank (Phylum, Class, Order, and Family) to compare sham and CCI injury groups at each timepoint. Genus and species level comparisons were not conducted due to increasing uncertainty for assigning a read to a genus or species with each progressive taxonomic rank.

Despite efforts to normalize the microbiome in all mice prior to CCI, our subsequent analysis detected a difference in the CCI and sham groups prior to injury. At the order level, *Erysipelotrichales* was more abundant at pre-injury baseline in the CCI group compared to the sham group (Supplemental Fig. 3). This suggests that our approach to microbiome normalization was largely effective but could not extinguish all pre-existing microbiome variation among the mice.

#### qPCR to assess *Akkermansia muciniphila*

Utilizing the same DNA isolated for 16s rRNA gene sequencing, qPCR was conducted using a custom TaqMan probe for *A. muciniphila* (Fwd: 5’ CGGTGGAGTATGTGGCTTAAT; Rev: 5’ CCATGCAGCACCTGTGTAA; Probe: 5’ CGCCTCCGAAGAGTTCGCATG; [20x]) and a 16 s pan-bacterial primer/probe set (Assay ID: Ba04930791_s1, ThermoFisher Scientific, # 4331182, [20x]). A 10 μl reaction was conducted with sample [DNA] = 5 ng/μl diluted as recommended by the manufacturer. Samples were run in triplicate on MicroAmp Fast Optical 96-well reaction plates (0.1 ml; # 4346907) and within-plate sample position was randomized. Reactions were carried out on a QuantStudio 7 Flex (Applied Biosystems) under the default parameters for use with Universal Master Mix.

Data output from qPCR were analyzed using the comparative CT (2^−∆∆Ct^) method^53^ to compare sham and CCI mice at 1, 2, and 3 d post-injury normalized to pre-injury. The ∆Ct value was derived by subtracting the mean Ct from the universal 16s probe from the mean Ct of the *A. muciniphila* probe for each sample (∆Ct = Ct_*A. muciniphila*_ − Ct_16s_), and the ∆∆Ct was derived by subtracting the ∆Ct_pre-injury_ from ∆Ct_post-injury_ (∆∆Ct = ∆Ct_post-injury_ − ∆Ct_pre-injury_).

### Statistical analyses

All analyses were performed in GraphPad Prism 9 unless otherwise specified with each animal considered to be an experimental unit. For statistical analysis of GI permeability data, sham mice from all timepoints were binned into a single sham group (n = 9) and compared against CCI mice that survived 4 h (n = 7), 8 h (n = 8), 1 d (n = 7), or 3 d (n = 6) by one-way ANOVA. Post-hoc analyses were conducted using Holm-Šídák's multiple comparisons test for each timepoint against sham controls. The reported group sizes reflect the exclusion of mice as detailed in Supplemental Table 1. Ileum crypt-villi, colon crypt, and Alcian Blue measurement data were assessed by two-way ANOVA followed by post-hoc Holm-Šídák's multiple comparisons tests. For hypoxia parameters, sham animals from both timepoints were binned for data analysis and a one-way ANOVA was performed followed by post-hoc Holm-Šídák's multiple comparisons tests. For all tests, α = 0.05. Model assumptions were checked by plotting the residuals on QQ plots.

Statistical comparisons for alpha diversity were conducted by mixed-effects ANOVA. Statistical analysis and graphing of beta diversity was conducted within R. Beta diversity ordination distances were compared by PERMANOVA (n_perm_ = 999) using the “adonis” function within the vegan package (v2.6-2 as described previously^45^). Post-hoc, pairwise comparisons were conducted using the “pairwise.adonis” function contained within the RVAideMemoire package (v0.9-81-2)^54^, and p-values were adjusted using the FDR by 2-stage linear step-up procedure of Benjamini, Krieger, and Yekutieli (Q:1%) for comparisons of interest for experimentally relevant comparisons only (n_perm_ = 999; FDR adj α = 0.05)^55^. Comparative CT data generated from qPCR output were analyzed by pairwise one-tailed t-tests with Welch’s correction to test for an increase in *A. muciniphila* in CCI mice compared to sham mice. For qPCR data, model assumptions were checked by plotting the residuals on QQ plots leading to linearization of the data using a log10 transform for statistical comparisons but were back-transformed for graphical presentation.

## Results

### Intestinal permeability

Although several studies have examined parameters related to intestinal permeability in rodent models of TBI, the majority have relied on ex vivo or invasive in situ permeability assays, measurement of permeability biomarkers in blood, and inferences drawn from measurements of tight junction protein levels^5,13,29,56–58^. We chose to employ an established, noninvasive method for assessing intestinal permeability in mice^30^ which has not yet been widely employed in neurotrauma models. Serum fluorescence was measured 4 h after oral gavage of 4 kDa FITC dextran. Under healthy conditions, paracellular permeability to FITC dextran within the GI tract should be minimal, leading to low serum fluorescence levels.

**Figure 2:**
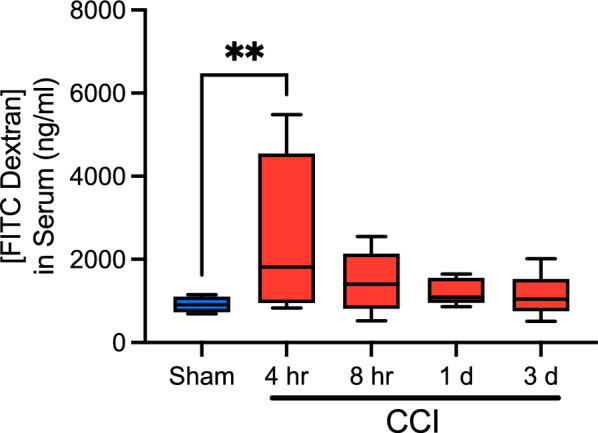
CCI results in a transient increase in intestinal permeability. Intestinal permeability was increased at 4 h following CCI when compared to sham animals binned from all timepoints. Serum concentration of FITC dextran returned to sham levels beginning at 8 h and remained there at 1 and 3 d post-injury. Whiskers represent minimum and maximum, box represents 25th-75th percentile, and line represents median value (**p < 0.01; n_sham_ = 2–3/timepoint, n_CCI_ = 6–9/timepoint). Data for individual sham groups can be found in Supplemental Fig. 4.

Permeability differed significantly among sham controls and mice euthanized at 4 h, 8 h, 1 d, and 3 d after CCI (Fig. 2; F(4,32) = 3.094; *p* = 0.03). Subsequent post-hoc comparisons of injured groups to the sham control group revealed increased GI permeability at 4 h after injury (*p* < 0.01), but not at 8 h, 1 d or 3 d post-CCI. These data are consistent with our interpretation that brain injury causes transient intestinal barrier dysfunction.

### Ileum and colon morphology

**Figure 3:**
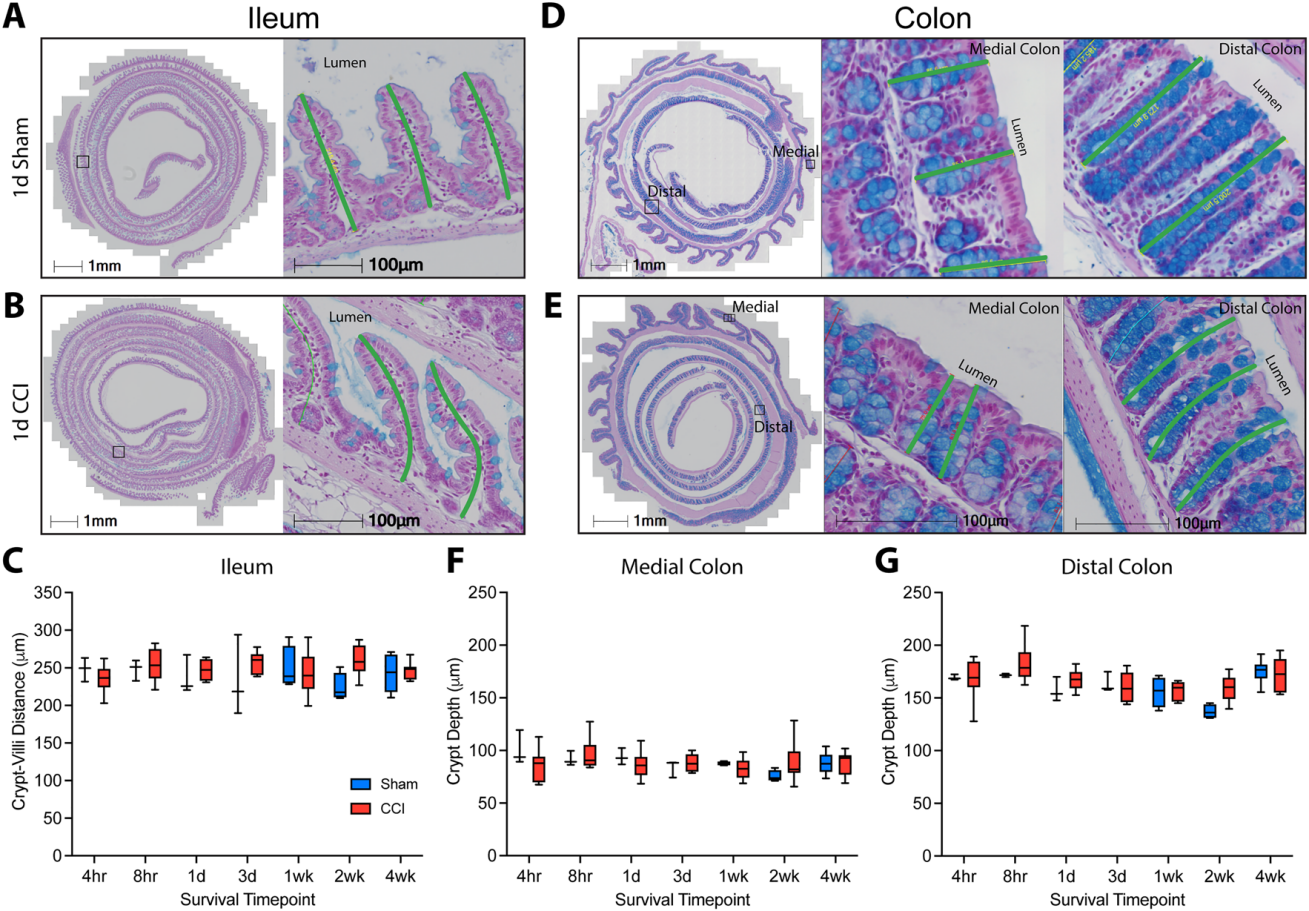
CCI does not alter morphology of the ileum or colon. Representative images of the crypt-villi structure from (**A**) sham and (**B**) CCI mice. Swiss-rolled cross sections are shown at low magnification with a box denoting the area shown at high magnification. Green lines depict the distance measurements acquired. (**C**) Quantification of crypt-villi depth for each timepoint shows no alteration in the ileum after CCI. Representative images illustrating crypt depth measurements in the colon (green lines) from (**D**) sham and (**E**) CCI mice. Quantification of the crypt depth suggests that CCI did not alter crypt morphology in the (**F**) medial or (**G**) distal colon. In the distal colon, a main effect of time after injury was present. Whiskers represent minimum and maximum, box represents 25th–75th percentile, and line represents median value (n_sham_ = 3–6/timepoint, n_CCI_ = 6–10/timepoint).

Changes in GI morphology have been reported in both the short and long term in various models of TBI^5,6,13,29^. Therefore, we assessed GI morphology in the ileum and colon over a comprehensive time course including 4 h, 8 h, 1 d, 3 d, 1 wk, 2 wk, and 4 wk after CCI or sham injury. Because alterations in the small intestine’s crypt-villi structure are indicative of damage^33,35,36^, villi morphology was assessed by measuring the crypt-villi distance in well-oriented regions of both sham (Fig. 3A) and CCI mice (Fig. 3B). Within the ileum, crypt-villi distance was not significantly altered by CCI (main effect of injury, F(1, 66) = 2.59, *p* = 0.11) and did not vary as a function of time post-injury (F(6, 66) = 0.21, *p* = 0.97; Fig. 3C). Further, no interaction was observed between injury and time post-injury (F(6, 66) = 1.33, *p* = 0.26).

Within the colon, shortened crypt depth is associated with acute inflammation while increased depth is associated with latent inflammation^34,59^. To assess whether CCI alters colon crypt morphology, the crypt depth was measured in the medial and distal regions of the colon for sham (Fig. 3D) and CCI mice (Fig. 3E). These two regions were quantified separately due to regional differences in function and crypt depth between the medial and distal portions of the colon. CCI had no overall effect on medial colon crypt depth (F(1,66) = 0.05, *p* = 0.82; Fig. 3F) or distal colon crypt depth (F(1,66) = 3.64, *p* = 0.11; Fig. 3G). No effect of time post-injury was observed in the medial colon (F(6,66) = 1.18, *p* = 0.33), but a main effect was present in the distal colon (F(6,66) = 5.87, *p* < 0.0001). However, no interaction was observed between injury and time in the medial colon (F(6,66) = 1.28, *p* = 0.28) or distal colon (F(6,66) = 1.29, *p* = 0.27), suggesting no overt morphological changes in the large intestine due to injury.

### Gut microbiome

#### Alpha diversity

**Figure 4:**
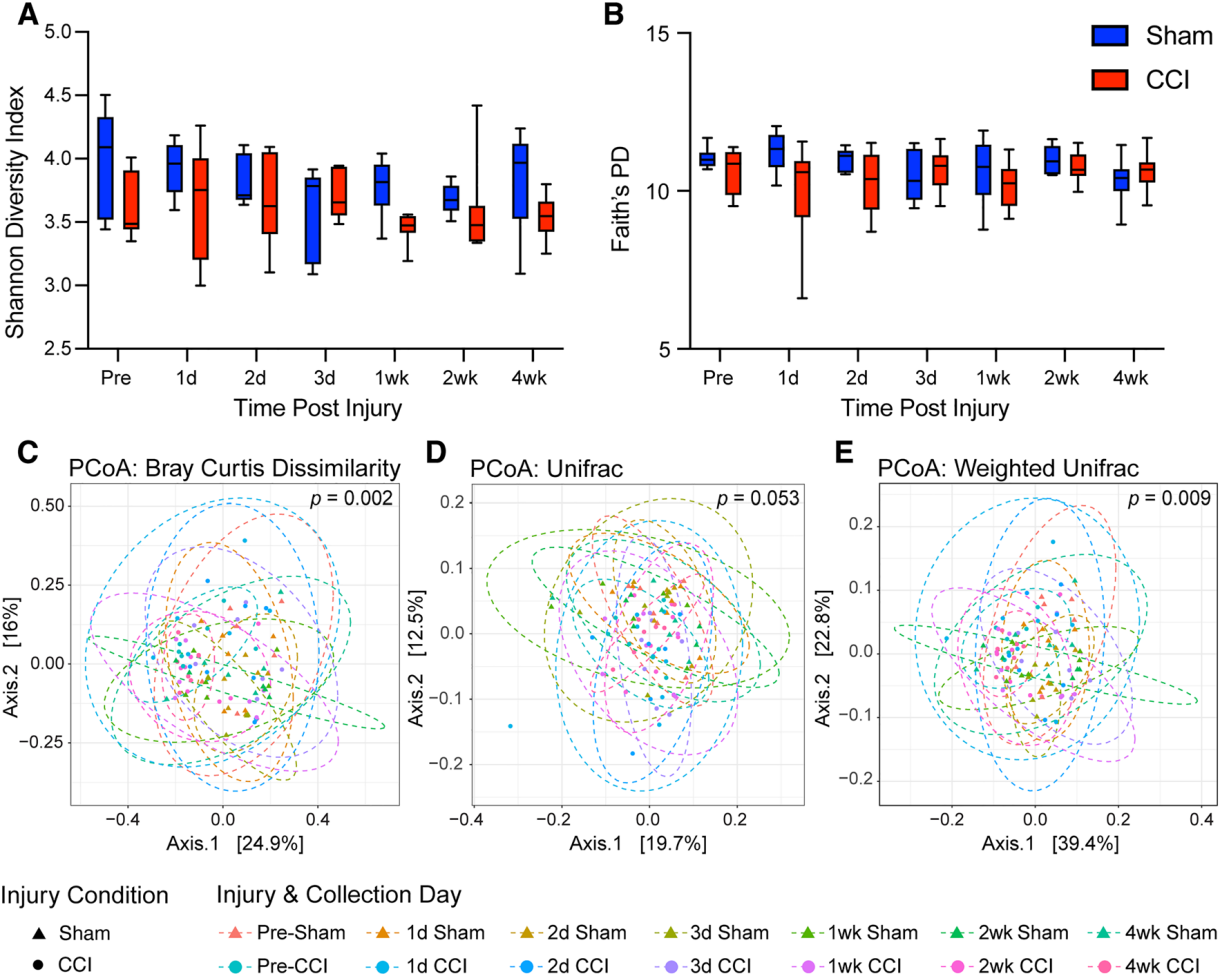
CCI results in decreased alpha diversity as measured by Shannon diversity index and altered beta diversity as measured by Bray–Curtis dissimilarity and weighted UniFrac distance. Alpha diversity: (**A**) the Shannon diversity index was significantly reduced in mice with CCI as compared to sham controls, but this injury effect did not depend on collection timepoint. (**B**) Faith’s phylogenetic diversity was not altered by injury or time point. Statistical comparison by mixed-effects analysis (whiskers represent minimum and maximum, box represents 25th–75th percentile, and line represents median value). Beta diversity: Principal coordinate analysis visualizations for (**C**) Bray–Curtis Dissimilarity (*p* = 0.002), (**D**) UniFrac distance (*p* = 0.053), and (**E**) weighted UniFrac distance (*p* = 0.009). Statistical comparison of Beta Diversity by PERMANOVA (n_sham_ = 6, n_CCI_ = 7).

To evaluate the hypothesis that TBI alters the microbiome, fecal samples were collected at multiple timepoints in mice surviving 4 wks following sham or CCI injury. Fecal microbial DNA was analyzed using 16s rRNA gene sequencing. Alpha diversity, a measure of within-sample diversity, was assessed by using Shannon Diversity Index and Faith’s phylogenetic diversity (PD). Shannon Diversity Index assesses within-sample diversity while accounting for richness (the number of observed taxa present) and evenness (the distribution of each taxon). Faith’s PD accounts for richness and evenness, as well as for phylogenetic distance between bacteria. Shannon diversity (Fig. 4A) was not altered with collection timepoint (F(3.32, 41.50) = 0.87; *p* = 0.47), but was reduced in the CCI group (F(1,75) = 8.03; *p* < 0.01) independent of time (F(1,75) = 1.08; *p* = 0.38). Faith’s PD (Fig. 4B) was not altered with collection timepoint (F(3.24, 34.53) = 0.44; *p* = 0.74), injury condition (F(1,11) = 3.20; *p* = 0.10), or their interaction (F(6,64) = 1.37; *p* = 0.24). A difference in Shannon diversity without a change in Faith’s PD suggests reduced richness and evenness within closely related phyla.

#### Beta diversity

To continue to investigate community level changes in the microbiome, we analyzed beta diversity, a between-sample comparison method used to determine the extent that bacterial profiles differ from each other. Beta diversity was assessed by three common measures: Bray–Curtis dissimilarity, UniFrac distance and weighted UniFrac distance. Beta diversity metrics have been reviewed by IMPACTT-investigators^60^. CCI induced a change in beta diversity when comparing groups for Bray–Curtis dissimilarity (*p* < 0.01; Fig. 4C), a well-established ecological calculation that provides a quantitative metric of how dissimilar two groups are from one another^61^. Additionally, CCI altered weighted UniFrac distance (*p* < 0.01; Fig. 4E) and there was a trend present with UniFrac distance (*p* = 0.053; Fig. 4D). UniFrac distance, weighted or unweighted, is a newer method of calculating differences between communities created specifically for 16 s rRNA gene sequencing data in which differences between groups are determined by shared taxa while accounting for phylogenetic distance^62^. After correcting for multiple-testing, post-hoc pairwise PERMANOVA for both Bray–Curtis and UniFrac showed no significant differences between comparisons of interest (Sham versus CCI at matched timepoints, and timepoint to timepoint comparisons within an injury group). Post-hoc comparison matrices are presented for Bray–Curtis dissimilarity within Supplemental Table 2 and for weighted UniFrac within Supplemental Table 3.

#### Differential abundance analysis

We next sought to identify differences in bacterial taxa between the CCI and sham-injured animals. To visualize these results, the relative abundance of each taxa at phylum and family levels was represented as a function of timepoint within each injury group (Supplemental Fig. 5). Consistent with prior studies^63,64^, large changes between injury and sham groups were not observed. The primary visual impression is that the effect of CCI on the microbiome is subtle with a potential difference being the phylum *Verrucomicrobiota* between sham and CCI mice (Supplemental Fig. 5). Within the *Verrucomicrobiota* phylum, the family *Akkermansiaeceae* shows a similar trend (Supplemental Fig. 5).

**Figure 5:**
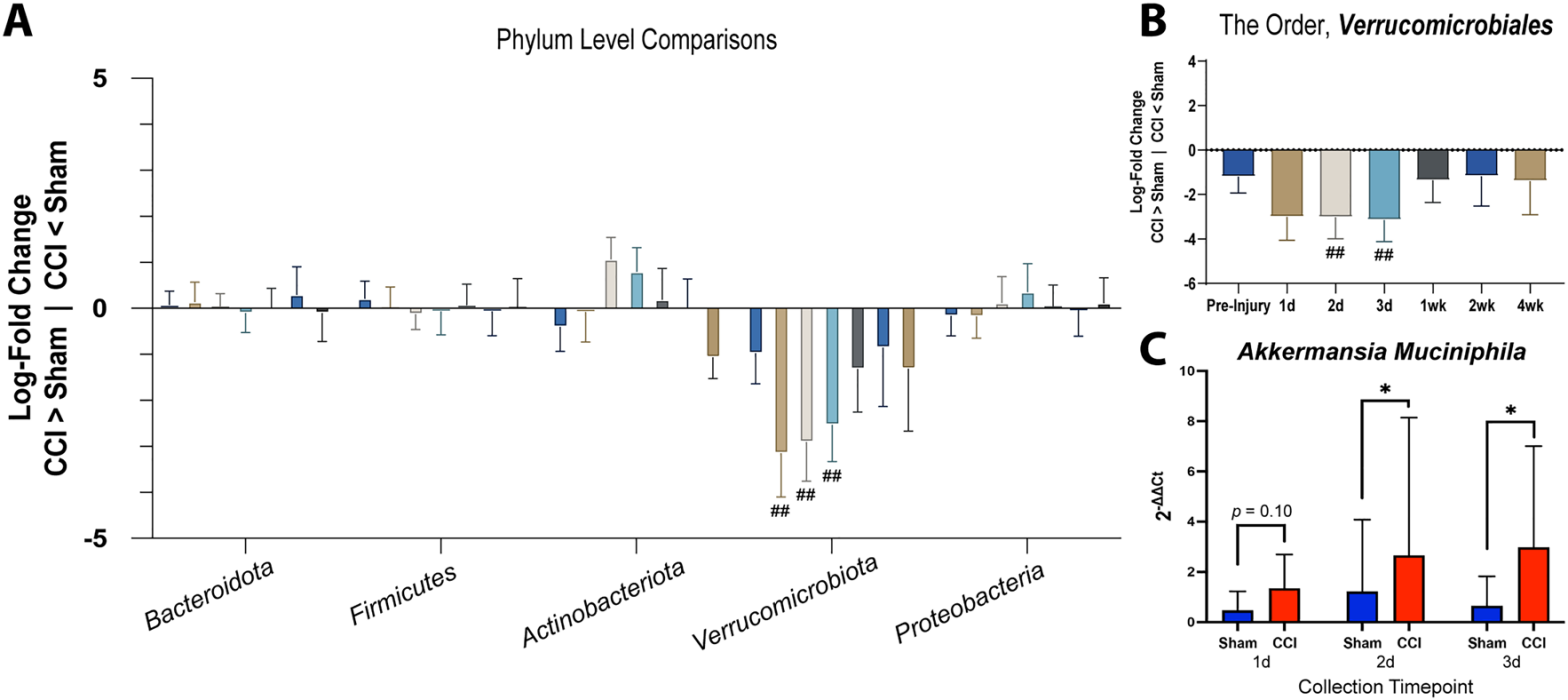
Early increase in abundance of taxa Verrucomicrobiota following CCI. (**A**) The phylum *Verrucomicrobiota* is differentially abundant in CCI animals compared to time-matched sham animals at 1, 2, and 3 d post-injury using ANCOM-BC. A log-fold change value (y-axis) of 0 indicates equivalent taxa abundance while above 0 indicates increased abundance in sham mice, and a log-fold change value below 0 indicates that a taxa is more abundant in CCI animals. (**B**) This taxa is differentially abundant down to an order level (*Verrucomicrobiales*) in CCI animals compared to time-matched sham animals at 2 and 3 d post-injury. Data are represented as log-fold change and standard error (## q < 0.01). (**C**) The *Verrucomicrobiota* species *Akkermansia muciniphila* is increased at 2 and 3 d post-injury as determined by qPCR. Data are represented as mean and standard deviation (*p < 0.05; n_sham_ = 6, n_CCI_ = 7).

To identify statistically significant differences in taxa between sham and CCI mice, we performed differential abundance analysis (ANCOM-BC) at phylum, class, order, and family taxonomic levels. The primary taxa altered after CCI was the phylum *Verrucomicrobiota*, which showed an early increase in CCI mice which was statistically significant at 1, 2, and 3 d post-injury when compared to time-matched sham animals (Fig. 5A). This increase was also statistically significant at the class level at these same time points (*Verrucomicrobiae;* data not shown) and at the order level at 2 and 3 d post-injury (*Verrucomicrobiales*; Fig. 5B).

The *Verrucomicrobiota* species typically residing in the mammalian GI tract is *Akkermansia muciniphila*. Because 16s sequencing does not provide sufficient resolution for consistent identification at the species level, we performed qPCR to directly assess whether *A. muciniphila* changed with CCI. We found that *A. muciniphila* was increased within the microbiome of injured mice at 2 d (*t*(8.47) = 1.92, p = 0.04, 95% CI [-0.18, 2.06]) and 3 d (*t*(8.12) = 1.90, *p* = 0.046, 95% CI [-0.23, 2.46]), with a trend at 1 d (t(10.86) = 1.38, *p* = 0.10, 95% CI [-0.31, 1.35]; Fig. 5C). These findings confirm that the increase in *Verrucomicrobiota* with CCI is due to an increase in *A. muciniphila*.

### Intestinal goblet cell quantification

Given that *A. muciniphila* lives within the mucus layer and is increased after CCI, we investigated the effect of CCI on ileal and colonic goblet cells. Mucus produced by goblet cells primarily acts as a lubricant while forming a selective barrier that limits epithelial interaction with luminal contents and bacteria^65^. Goblet cells also produce anti-microbial peptides that shape the microbiome^66–68^. Mucus contained within goblet cells was labeled using the muciniphilic dye, Alcian Blue.

**Figure 6:**
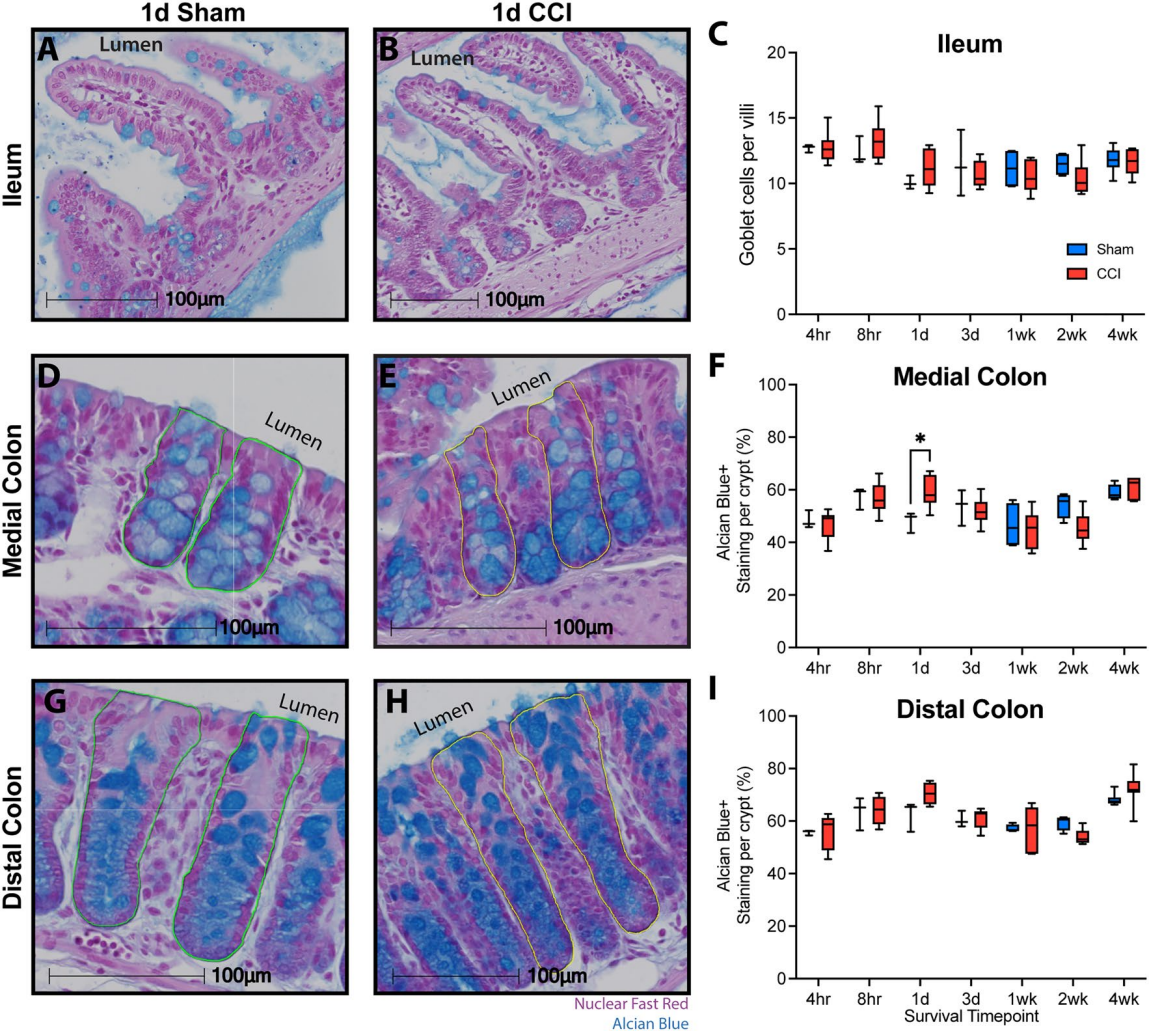
Goblet cells are altered in the medial colon but not in the ileum or distal colon. Intestinal tissue was stained with Alcian Blue to label mucins within goblet cells and counterstained with nuclear fast red. Representative images of the distal third of the small intestine, the ileum, from (**A**) 1 d sham and (**B**) 1 d CCI mice. (**C**) Counts of goblet cells per villi were equivalent for sham and CCI groups but varied across timepoints. Representative images of colon tissue from both the (**D, E**) medial and (**G, H**) distal colon of 1 d sham and 1 d CCI mice, respectively. (**F**) CCI increased goblet cell density in the medial colon at 1 d, (**G**) but no effect of injury was observed in the distal colon. A main effect of time post-injury was present in both regions of the colon. Whiskers represent minimum and maximum, box represents 25th–75th percentile, and line represents median value (*p < 0.05; n_sham_ = 3–6/timepoint; n_CCI_ = 6–10/timepoint).

CCI did not alter the number of goblet cells within the ileum (main effect: F(1,66) = 0.10; *p* = 0.76; Fig. 6A–C). Goblet cell number varied as a function of time post-injury (F(6,66) = 4.92; *p* < 0.001), but this effect did not depend on injury status (F(6,66) = 1.05; *p* = 0.40). Goblet cell density was assessed in the medial (Fig. 6D–F) and distal (Fig. 6G–I) regions of the colon. Within the medial colon a significant interaction between injury condition and time post-injury was observed (F(6,66) = 2.65; *p* = 0.02) with a significant main effect of timepoint (F(6,66) = 8.94; *p* < 0.0001), but no main effect of injury (F(1,66) = 0.01; *p* = 0.92). Post-hoc comparisons revealed a significant increase in goblet cell density at 1 d in CCI mice compared to sham mice (Fig. 6F; *p* = 0.03). Within the distal colon, goblet cell density was not altered by injury condition (F(1,66) = 0.82; *p* = 0.38), but varied across timepoints (F(6,66) = 12.93; *p* < 0.0001) without a significant interaction (Fig. 6I; F(6,66) = 1.67; *p* = 0.14).

### Colon hypoxia

**Figure 7:**
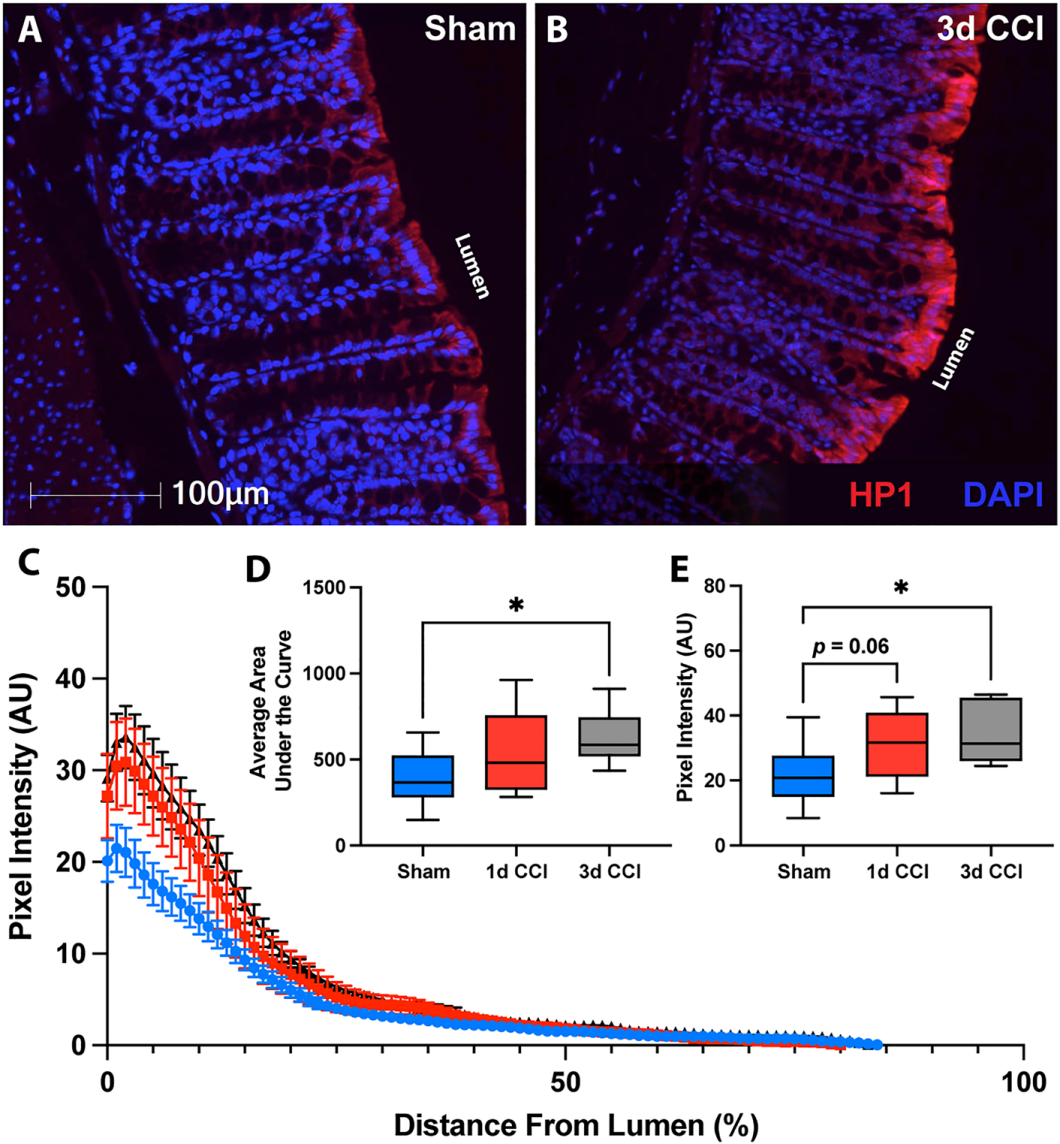
CCI induces colon hypoxia at 3 d post-injury. Representative images of hypoxyprobe-1 staining (HP1, red) in the colon of (**A**) sham and (**B**) 3 d post-CCI mice. (**C**) Traces of hypoxia intensity as a function of distance from the lumen, averaged from three crypts within three randomly selected fields of view from sham (blue), 1 d CCI (red), and 3 d CCI (black). (**D**) Area under the hypoxia intensity curve is increased at 3 d post-CCI compared to sham mice. (**E**) Peak hypoxia is also increased at 3 d post-CCI. Whiskers represent minimum and maximum, box represents 25th–75th percentile, and line represents median value (*p < 0.05; n_sham_ = 11, n_CCI_ = 6–7/timepoint).

To further investigate the means by which *A. muciniphila* may be increasing after CCI we assessed colon hypoxia using pimonidazole-HCl in sham (Fig. 7A) and CCI mice (Fig. 7B). Because *A. muciniphila* is an obligate anaerobe and lives in relatively close association with the host epithelium, its abundance has been shown to increase under hypoxic conditions. For example, Alam et al.^69^ showed that wound-induced hypoxia results in a preferential increase of *A. muciniphila* at the site of hypoxia. These microbes then in turn promote epithelial contributions to wound healing^69^.

Under healthy circumstances, the colon exists in a state of physiologic hypoxia. As cells migrate out of the crypt stem-cell niche toward the lumen they shift toward oxidative phosphorylation which leads to decreased partial pressure of oxygen. As such, luminal facing epithelial cells exist in a state of hypoxia^39^. Importantly, epithelial injury in conditions such as colitis leads to reduced mitochondrial oxygen utilization and diminished intestinal epithelial hypoxia^70^. Disturbances in oxygen distribution can alter the degree of hypoxia as well as its penetration within the crypt. Immunolabeling for pimonidazole-HCl protein adducts formed under hypoxic conditions allows for visualization and quantification of the hypoxic gradient within the colon via analysis of staining intensity as a function of distance along the luminal-basal axis (Fig. 7C). Following CCI, hypoxia appeared more intense with deeper penetration into crypt structures. Indeed, both the area under the curve (F(2,21) = 3.64; *p* = 0.04) and peak intensity (F(2,21) = 4.1; *p* = 0.03) of hypoxyprobe-1 labeling revealed progressive increases over the first days after CCI. Area under the curve showed a significant increase at 3 d (*p* = 0.03; Fig. 7D) while peak hypoxic intensity showed a trend toward an increase at 1 d (*p* = 0.06) and was significantly elevated at 3 d post-injury (*p* = 0.03; Fig. 7E). Colon hypoxia was not notably altered at the 4 h timepoint that corresponds with increased intestinal permeability (Supplemental Fig. 6).

## Discussion

In this study, we assessed effects of a moderate severity contusive brain injury on GI tract barrier function and morphology, the fecal microbiome and colon hypoxia. Using a noninvasive FITC dextran permeability assay, intestinal permeability was found to increase at 4 h after CCI injury, returning fully to the level of sham controls by 1 d. To determine if this early change in permeability contributed to tissue damage along the GI tract, we evaluated small and large intestinal morphology. CCI did not alter ileal crypt-villi distance or colon crypt-depth at the time of the permeability increase or at subsequent timepoints spanning a month after the injury. Fecal microbiome analyses revealed a reduction in Shannon diversity in the CCI group and alterations in beta diversity between groups. Differential abundance analysis identified increases in the phylum Verrucomicrobiota at 1, 2, and 3 d post-injury. The *Verrucomicrobiota* species, *A. muciniphila*, was confirmed by qPCR to increase over the same time. To assess physiological factors known to either coincide with or propagate growth of this obligate anaerobe, mucus-producing goblet cells and colonic hypoxia were evaluated. CCI led to increased goblet cell density in the medial colon at 1 d post-injury but did not affect goblet cells within the ileum or distal colon. In addition, CCI promoted an early increase in colon hypoxia that was statistically significant by 3 d post-injury.

Several previous studies support that TBI increases permeability of the GI tract. Clinically, increased permeability of the small intestine can be detected by an increased lactulose: mannitol ratio in the urine after oral administration of these two non-metabolized sugars^71^. This approach has also been used to infer TBI-related increases in gut permeability in rats from 3 h to 1 wk after injury^29,58^. Its interpretation may not be straightforward, as an elevated lactulose: mannitol ratio can result from either increased paracellular transport of lactulose (i.e. increased permeability) or a decrease in mannitol due to loss of absorptive area in the gut consequent to damage to ileal villi structures^29^. Increased paracellular permeability after brain injury is believed to be due to loss or dysfunction of tight junction proteins that regulate paracellular flux. Reductions in several tight junction proteins have been demonstrated following rodent experimental TBI in the ileum at 3–12 h^5,57^ and 3 d^72^, in the cecum at 3 d^73^, and in the proximal colon at 2 wk post-injury^74^.

Here, we found only a transient increase in GI permeability at 4 h using a noninvasive intestinal permeability assay to measure paracellular movement of FITC dextran from the intestinal lumen into the bloodstream. The brief nature of the alteration in permeability in our study as compared to more sustained dysfunction reported in other studies may be due, in part, to differences in injury severity, injury model, or permeability assay. Notably, prior studies that identified increased FITC dextran permeability up to 12 h after weight drop injury in mice^5,13,56^ or rats^57^ used an invasive approach in which the intestinal tract is removed from the abdominal cavity, ligated and injected with FITC dextran before being returned to the body cavity. These manipulations may have contributed to a more protracted permeability disruption.

Changes in intestinal barrier function often correspond with changes to the specialized crypt-villi and crypt structures that line the small and large intestine, respectively. Contusion TBI in rodents has been reported to induce structural damage in the ileum. However, many prior studies have focused on a single region of the GI tract and a single timepoint after injury, utilizing qualitative or semi-quantitative assessments. Here, we performed a quantitative histological examination of ileal crypt-villi distance and colon crypt depth over an extended time course. We found that CCI did not induce changes to the crypt-villus length in the small intestine or crypt depth in the colon at any timepoint. These data contrast findings of epithelial lifting^29,57^ and either villus lengthening^12^ or shortening^13^ within the first several hours, and decreases in villus length and crypt depth over the period from 1 to 7 d post-injury^29,58,72^. A potential caveat to several of these studies is that postmortem histological examinations were performed on segments of the ileum that had previously been externalized from the mouse or rat, ligated and injected with FITC dextran to assay GI permeability^5,13,56,57^. Thus, tissue ischemia, hypoxia or inflammation resulting from this procedure may have exaggerated effects of the TBI. As the majority of studies noting GI tissue pathology employed injury parameters described as creating a severe TBI^12,29,57,72^, overt GI histological damage may be a consequence of severe, rather than moderate, experimental TBI. For example, images of brain histology provided in Ma, Y. et al.^72^ demonstrate loss of not only of cortex but also the underlying hippocampus, while the moderate CCI employed here results in a contusion localized to the cortex, leaving the hippocampus grossly intact (see Fig. 1). Few studies have examined histopathology within the colon after TBI. Following severe CCI, edema was reported in some rats at 6 h^12^, but no damage was detected at 3 d^73^. While Ma, E. et al.^6^ found no histological damage in the colon at 1 d consistent with our findings, they noted increased crypt depth and smooth muscle layer thickness at 28 d, suggestive of delayed or progressive damage. Our data argue that moderate CCI is not associated with overt histological damage to the small or large intestine over the first 4 wk after injury. It is possible, however, that small, localized regions of shortening/lengthening were not appreciated due to the large number of crypt-villi and crypt structures we assessed along the longitudinal axis of the Swiss-rolled tissue preparations.

Mice with CCI had reduced gut microbiome alpha diversity compared with sham controls, as measured by Shannon Diversity. This effect was not dependent upon timepoint after injury but is consistent with observations of reduced alpha diversity in previous studies^73,75^. Microbiome composition differed among experimental groups with respect to beta diversity, with most notable differences in the 4 wk post-injury group, in line with late changes in beta diversity reported in other studies of CCI^75^.

Changes in the compositional abundance of the gut microbiome after TBI are complex and vary across studies, as recently reviewed by Hanscom, Loane and Shea-Donohue^20^. Here, moderate CCI resulted in relatively limited alterations in the microbiome, with significant increases in taxa within the phylum *Verrucomicrobiota* at 1, 2 and 3 d. The lack of widespread changes may be due in part to stringent efforts to minimize variation in pre-injury microbiome composition. We first cleared the microbiome with PEG before inoculating mice with a common microbiome collected from the cecal contents of age- and sex-matched mice and then used a mixed bedding approach to maintain similar microbiomes prior to injury. As mentioned above, the use of moderate, rather than severe, injury may also have resulted in more limited changes to the microbiome than previously reported. Increases in the phylum *Verrucomicrobiota* have been observed in mice^75,76^, rats^64,72^ and humans^77^ after brain injury, affirming the robustness of this observation across species and injury model.

Although the major gut microbe within this phylum is *A. muciniphila*, our study is the first to our knowledge to utilize qPCR to confirm that the *Verrucomicrobiota* species increased after TBI is *A. muciniphila*. This species is a gram-negative bacteria that is most prevalent in the colon where it resides in the intestinal mucus layer^78^. *A. muciniphila* is critical to maintaining normal gut health^79^, with alterations in abundance influencing intestinal epithelial growth, differentiation^80^, integrity^81^, and wound healing^69^. An early increase in *A. muciniphila* may be part of a compensatory response to systemic damage initiated by TBI. Recently, *A. muciniphila* levels were shown to be reduced at 1 wk and 4 wk in mice receiving CCI of a similar severity to the present study^82^, suggesting that trauma-induced changes in *A. muciniphila* may be biphasic.

*A. muciniphila* feeds off of the intestinal mucus layer and, by doing so, signals to the host to increase and maintain mucus production^83^ while also influencing intestinal stem cell differentiation and subsequent outgrowth^80^. Based on the important role that *A. muciniphila* plays in maintaining the intestinal mucus layer, we investigated changes to the mucus-producing goblet cells. Numbers of goblet cells in the ileum were unchanged with moderate CCI. However, goblet cell density increased within the medial colon at 1 d post-injury, with no change in the distal colon. Transverse folds of the medial colon may provide a more stable mucosal niche for bacteria than in the distal region of the colon^84^. An increase in density could reflect increases in goblet cell number or size. The goblet cell response to inflammation is complicated in that, under most inflammatory conditions, goblet cell differentiation factors generally increase, leading to greater numbers of goblet cells^85^. Some inflammatory conditions such as ulcerative colitis, however, are associated with a reduction in the number and size of goblet cells^86,87^. Only one other study has assessed goblet cells after brain injury, finding no difference in the cecum at 3 d after a mild closed head injury^88^. As the mucus layer plays a key role in barrier function and mediates numerous host-microbe interactions, future studies should consider assessing changes to the mucus layer thickness and composition after TBI.

Both bacterial products and epithelial metabolism can alter oxygen availability within the intestinal lumen^39,89^. Our findings show that CCI leads to a progressive increase in colon hypoxia over 3 d following injury. Together with the increase in goblet cell density at 1 d, we hypothesize that posttraumatic hypoxia leads to conditions within the colon that promote *A. muciniphila* proliferation. As an obligate anaerobe, *A. muciniphila* thrives in hypoxic conditions present in the healthy colonic GI tract. Due to the relatively close association of *A. muciniphila* with the epithelium, hypoxic conditions within the epithelium and lumen can promote the growth of this obligate anaerobe^69^.

Colon hypoxia following TBI may provide mechanistic insight as to how microbe-epithelial interactions are altered after TBI. Under homeostatic conditions within the colon, the microbiota promote anaerobic luminal conditions that support *Firmicutes* and suppress host inflammation^90^. Some anaerobic bacteria, such as *A. muciniphila*, produce butyrate, a short chain fatty acid that is a major fuel source for the host epithelium^91–93^. Processing of butyrate through mitochondrial β-oxidation to produce ATP is an oxygen-intensive process that results in the physiologic hypoxic gradient^89^. Increased colon hypoxia after TBI may reflect elevated oxidative phosphorylation in epithelial mitochondria. *A. muciniphila* produces both acetate and butyrate in vitro when grown in both human and porcine mucins^91^, so it is possible that elevated *A. muciniphila* generates a localized increase in butyrate that stimulates further mitochondrial β-oxidation. Therefore, increased goblet cell density in the proximal colon may support greater butyrate generation and potentiate mitochondrial mediated hypoxia. In models of colitis, supplementation with butyrate attenuates colon inflammation and histological damage^94^. Additional experiments are needed to address butyrate levels and the role of epithelial mitochondrial oxidative phosphorylation in TBI. Cecal butyrate concentration did not change following severe CCI^75^, but severe injury may preclude the endogenous compensatory response observed in this study of moderate CCI. However, we did not observe significant changes in *Clostridia*, the class most commonly associated with butyrate production^92^, suggesting other circulating factors such as cortisol may stimulate epithelial oxygen utilization^95^. The cellular and molecular responses triggered by an early increase in GI permeability that initiate the potentially reparative increases in A. muciniphila, goblet cell density and colon hypoxia are yet to be elucidated.

From a potential treatment perspective, others have shown beneficial effects of direct administration of *A. muciniphila* as a means of treatment for neurological conditions in rodent models of depression^96^, Alzheimer’s disease^97^, and amyotrophic lateral sclerosis^98^. Additionally, dietary supplementation of the fermentable fiber inulin increases *A. muciniphila* and was found to increase thalamic and hippocampal blood flow after closed head TBI^76^, suggesting that inulin and perhaps *A. muciniphila* are beneficial after TBI. More broadly, fecal *A. muciniphila* levels have been proposed as a marker for discriminating between healthy controls and patients with both intestinal and extra-intestinal conditions^99^.

We acknowledge that this study has several limitations. Regarding experimental design, only male mice were utilized in these studies. Very few TBI studies assessing changes to the GI tract or microbiome have included female mice^100,101^ which may limit translatability of findings. Additionally, not all experimental procedures were carried out in all mice which limits the ability to directly correlate outcomes. Further, only mice from the 4 wk timepoint underwent microbiome normalization and injury condition-specific group housing. Although we saw no morphological damage at any time point, it is possible that these conditions affected histological parameters at 4 wk as compared to those measured at earlier time points in mice with a naturally occurring microbiome. Despite our efforts to standardize the microbiota, there were still minor differences detected at baseline by differential abundance analysis. Differences in microbiota prior to injury could alter the intestinal response to brain injury. It is possible that overnight fasting prior to gavage with FITC dextran may have had a more pronounced effect upon intestinal permeability than daytime fasting. Future studies could standardize fasting relative to the light/dark cycle for assessment of intestinal permeability.

## Conclusions

**Figure 8:**
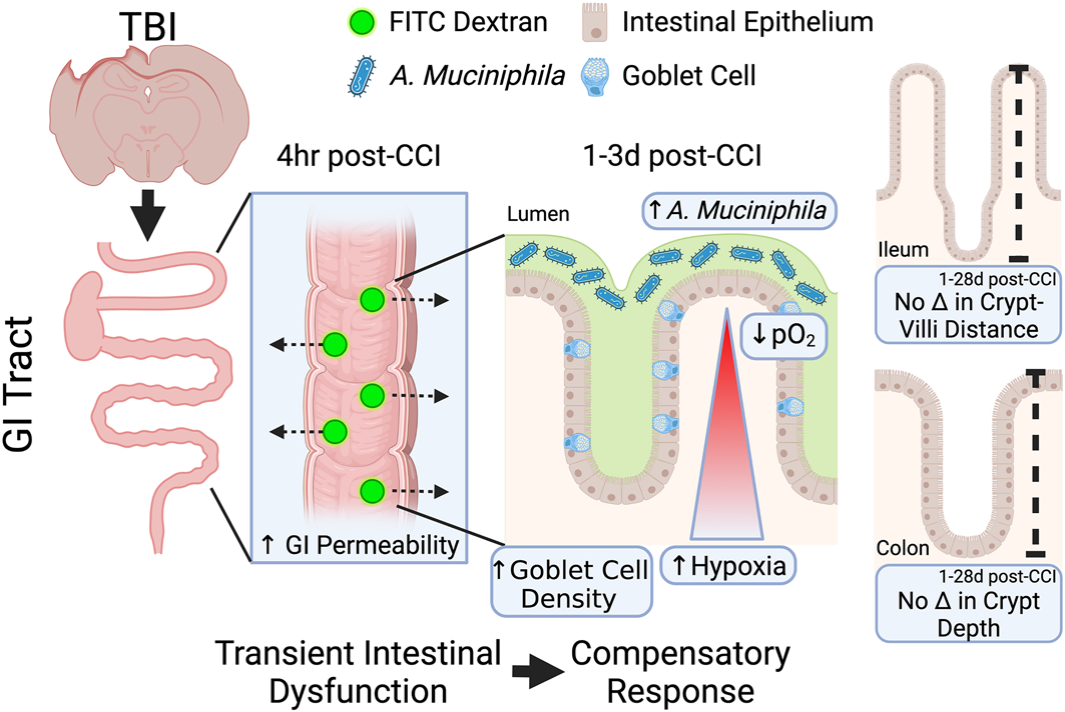
Graphical summary of findings. We hypothesize that TBI leads to a transient disruption of the intestinal barrier that initiates a colonic response characterized by increased goblet cell density and hypoxia, facilitating an increase in *A. muciniphila* as a beneficial compensatory response to limit damage to the GI tract. Figure created with BioRender.com.

Overall, our findings add to the field’s understanding of the complexity of the GI tract’s response to TBI. Our current working hypothesis is visually summarized in Fig. 8. Our primary findings are that TBI induces an early, transient increase in intestinal permeability and a bloom of *A. muciniphila* that corresponds temporally with increased medial colon goblet cell density and increased colon hypoxia in the absence of altered GI tract morphology. Since this bacterium has been associated with improved barrier integrity and general gut health, the increase in *A. muciniphila* with TBI may reflect a beneficial compensatory alteration in the gut microbiome sufficient to limit histological damage to the GI tract after moderate TBI. Because of the unique role of *A. muciniphila*, potential interventions that prolong or promote the growth of this bacteria warrant further investigation as novel therapeutics after TBI.

### Supplementary Information

Supplementary Information.

## Data Availability

Reporting on methodology, analysis and results are in accordance with ARRIVE guidelines^102^. 16s rRNA gene sequencing data generated and/or analyzed during the current study are available in the Gene Expression Omnibus repository under accession number GSE239472 (https://www.ncbi.nlm.nih.gov/geo/query/acc.cgi?acc=GSE239472). All other datasets used and/or analyzed during the current study are available from the corresponding author on reasonable request.

## Abbreviations

ANCOM-BC: Analysis of Compositions of Microbiomes with Bias Correction
ANOVA: Analysis of variance
ASV: Amplicon Sequence Variants
CCI: Controlled Cortical Impact
d: Day(s)
DADA2: Divisive Amplicon Denoising Algorithm
ddH_2_O: Double distilled water
DNA: Deoxyribonucleic acid
FDR: False discovery rate
FITC: Fluorescein isothiocyanate
GI: Gastrointestinal
HCl: Hydrochloride
HP1: Hypoxyprobe-1 (brand name for pimonidazole-HCl)
hr: Hour(s)
i.p.: Intraperitoneal
kDa: Kilodalton
mAb: Monoclonal antibody
min: Minute(s)
NCBI: National Center for Biotechnology Information
NHS: Normal horse serum
pAb: Polyclonal antibody
PBS: Phosphate-buffered saline
PEG: Polyethylene glycol
qPCR: Quantitative polymerase chain reaction
rmANOVA: Repeated measures ANOVA
rRNA: Ribosomal ribonucleic acid
TBI: Traumatic brain injury
TBS: Tris-buffered saline
TBS-T: 0.1% Triton X-100 in 1 × TBS
wk(s): Week(s)
