# Supplementary material for: Acute gastrointestinal permeability after traumatic brain injury in mice precedes a bloom in Akkermansia muciniphila supported by intestinal hypoxia
Source: Sci Rep. 2024 Feb 5;14:2990. doi: 10.1038/s41598-024-53430-4 (PMC10844296; doi:10.1038/s41598-024-53430-4)
Supplement: Supplementary file 1 — Supplementary Information. [file 41598_2024_53430_MOESM1_ESM.docx]

# ­­Supplemental Information

# Acute gastrointestinal permeability after traumatic brain injury in mice precedes a bloom in Akkermansia muciniphila supported by intestinal hypoxia

Anthony J. DeSana^1,2^, Steven Estus^1,5^, Terrence A. Barrett^3,4^, and Kathryn E. Saatman^1,2*^

^1^Department of Physiology, University of Kentucky, Lexington, KY

^2^Spinal Cord and Brain Injury Research Center, University of Kentucky, Lexington, KY

^3^Department of Internal Medicine – Digestive Health, University of Kentucky, Lexington, KY

^4^Department of Microbiology, Immunology & Molecular Genetics, University of Kentucky, Lexington, KY

^5^Sanders Brown Center on Aging, University of Kentucky, Lexington, KY

*Corresponding author


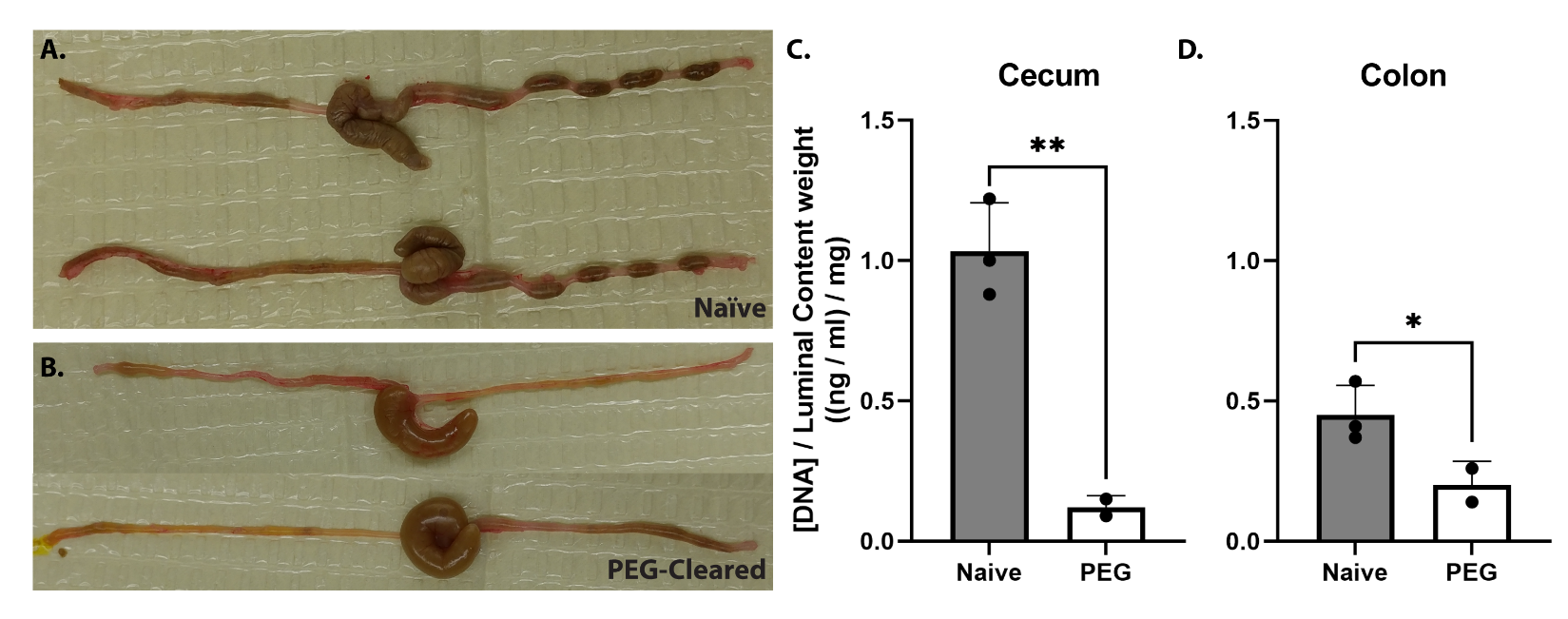


Supplemental Figure 1: Whole bowel irrigation with PEG effectively reduces the concentration of DNA in the luminal contents of the large bowel

Image of the terminal segment of the small intestine, cecum, and colon excised from **(A)** a naïve animal and **(B)** a mouse that underwent whole bowel irrigation via oral gavage of polyethylene glycol. The concentration of DNA within the luminal contents is significantly reduced in the **(C)** cecum and **(D)** colon. Data represent individual mice as well as mean and standard deviation (one-tailed T-test; **p <* 0.05, ***p* < 0.01; n_naive_ = 3, n_PEG_ = 2).


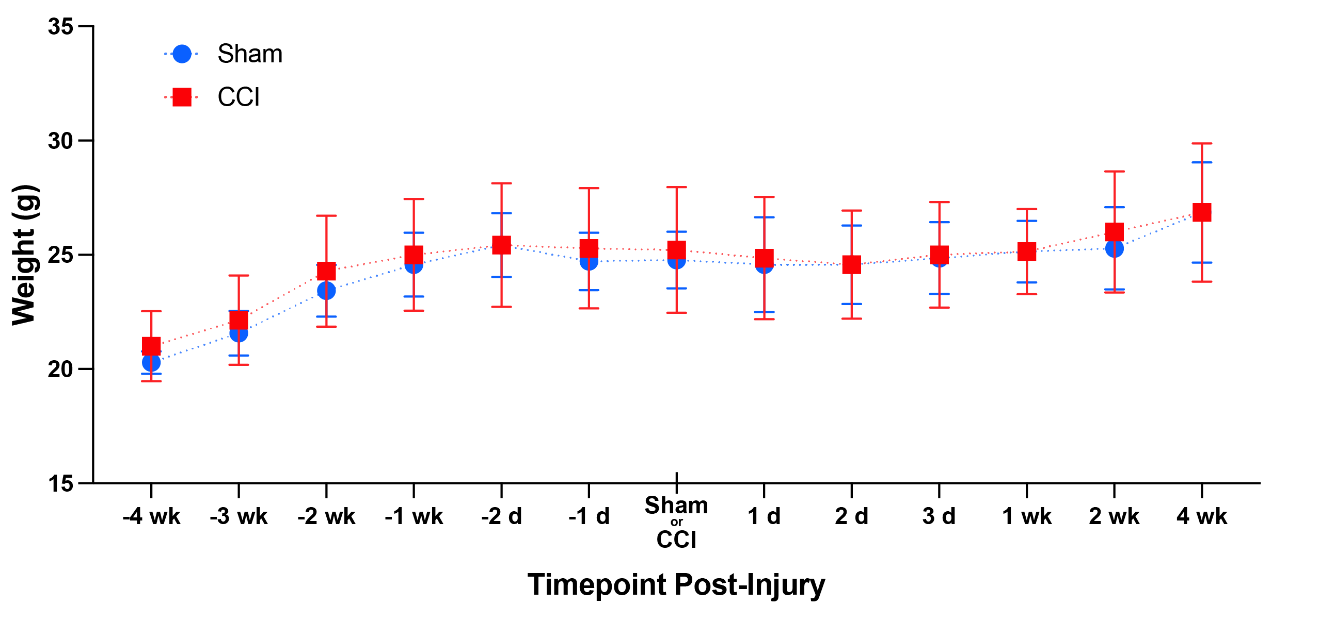


Supplemental Figure 2: Body weight did not differ between injury groups prior to or after injury

Mice were weighed at each inoculation prior to injury, at 1 and 2 d prior to injury, on the day of sham/ CCI surgery, and then again with each fecal collection out to 4 wk post-injury. There was no significant effect of injury condition (F(1, 12) = 0.13; *p* = 0.73), and a main effect of time was observed (F(3.84, 46.12) = 74.40; *p* < 0.0001) without an interaction (rmANOVA; F(12, 144) = 0.70; *p* = 0.75). Data represent mean and standard deviation (n_sham_ = 6, n_CCI_ = 7).


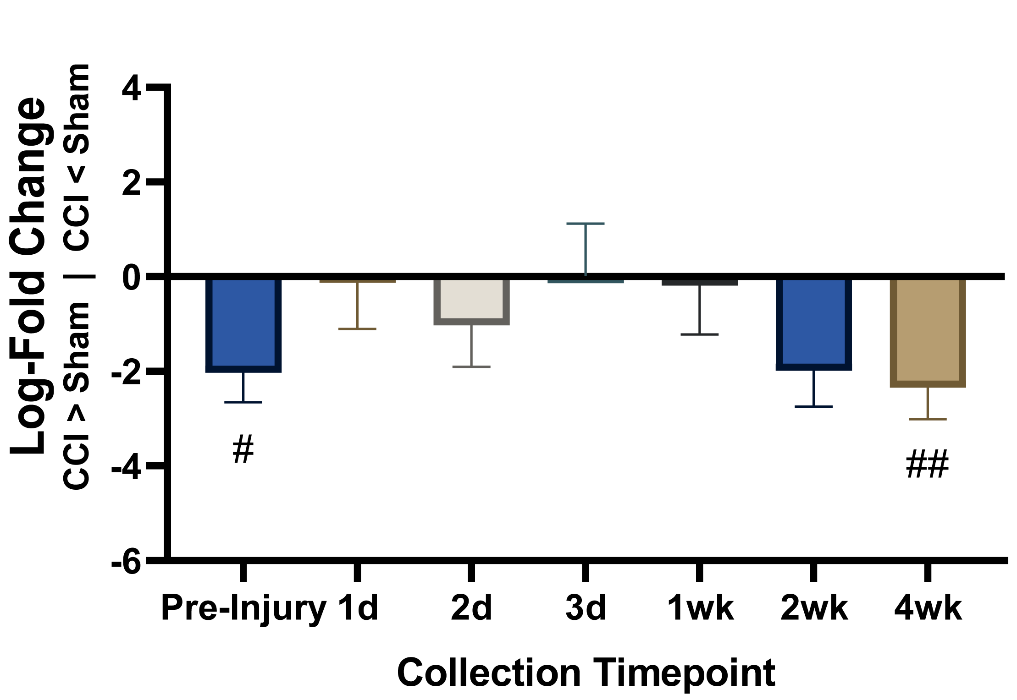


Supplemental Figure 3: The order *Erysipelotrichales*

Prior to injury the order *Erysipelotrichales* was differentially abundant in mice that would receive a CCI, and this difference returned at 4 wk post-injury. Data are represented as log-fold change and standard error (ANCOM-BC; # *q* < 0.05, ## *q* < 0.01; n_sham_ = 6, n_CCI_ = 7).


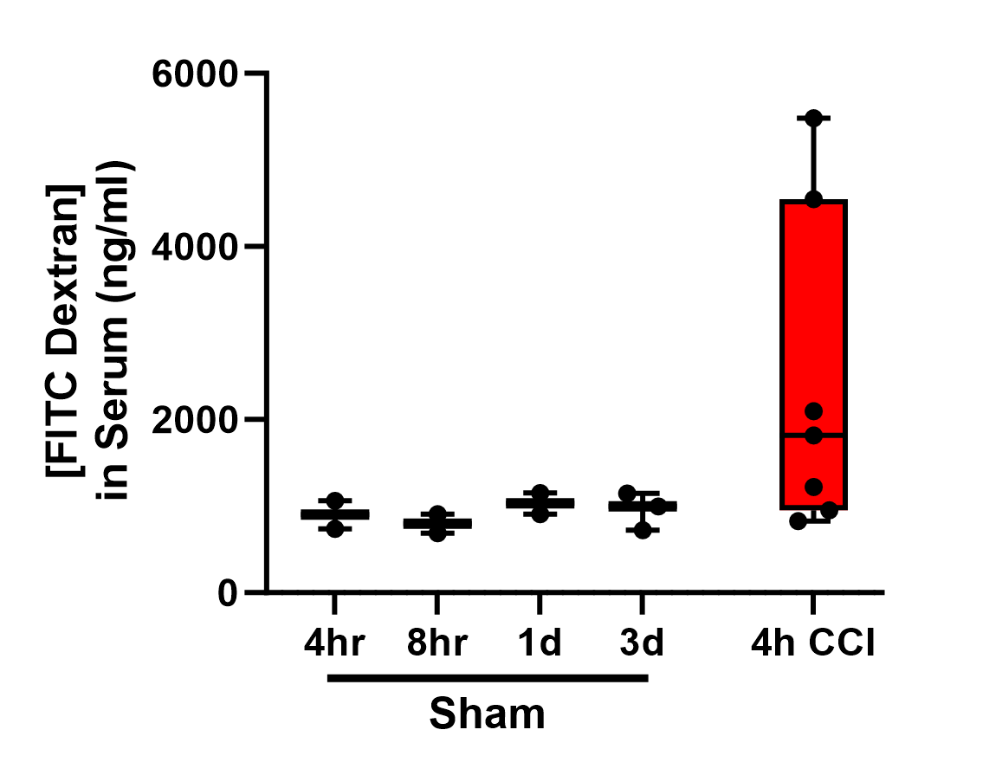


Supplemental Figure 4: Permeability responses for individual sham groups in reference to the 4 h CCI group

There was minimal variability in intestinal permeability across sham groups regardless of timepoint. Sham animals were intended to be binned as part of the experimental design. As such, this figure is for visualization of group variability and no statistics were performed. Sham data represent individual mice as well as mean and standard deviation (Shams: n_4h_ = 2, n_8h_ = 2, n_1d_ = 2, n_3d_ = 3; CCI_4h_ = 7).


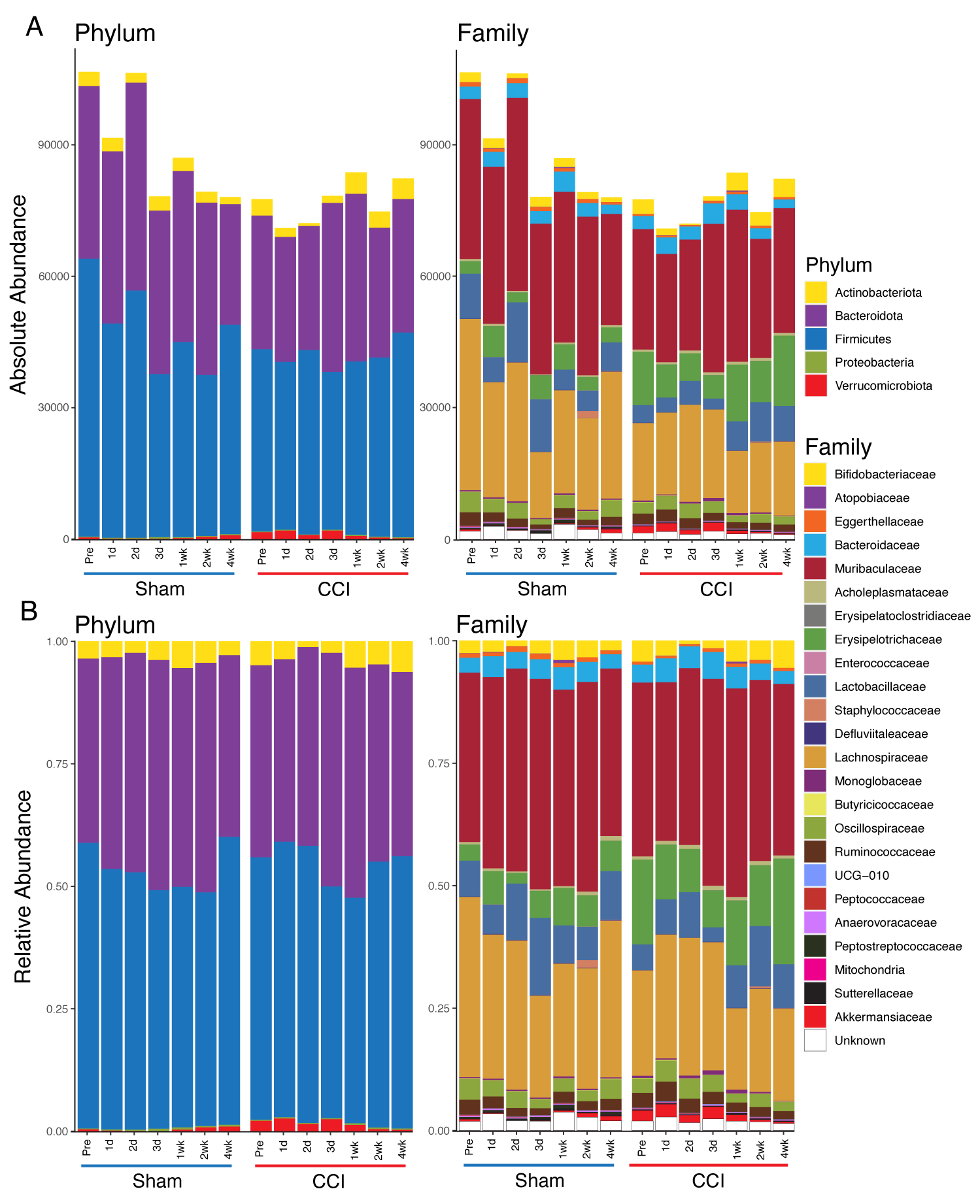


Supplemental Figure 5: Absolute and relative abundance taxa distribution

Representation of **(A)** absolute and **(B)** relative abundance by injury group and collection timepoint at a phylum and family level (n_sham_ = 6, n_CCI_ = 7).


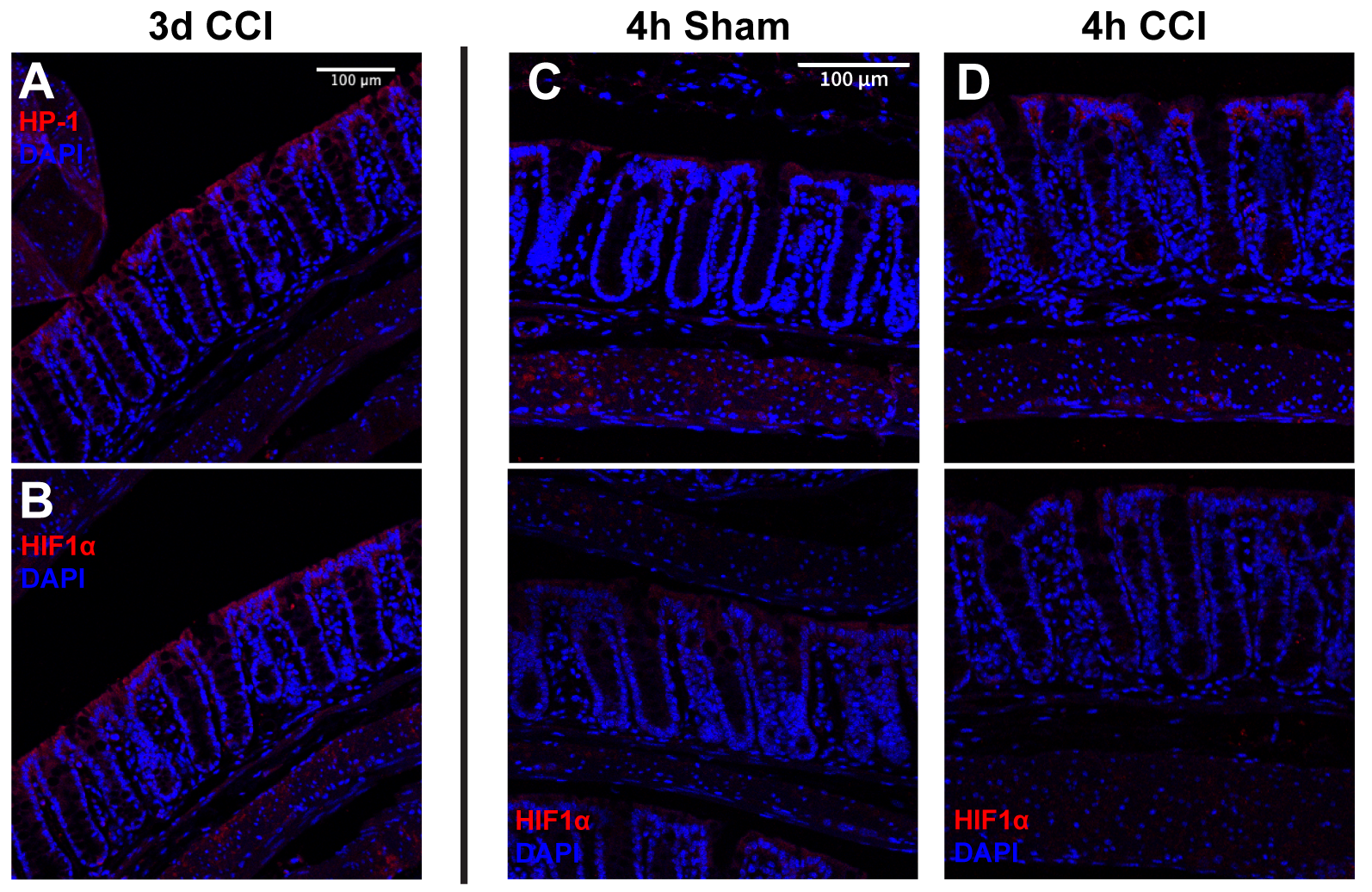


Supplemental Figure 6 : Colon HIF1α labeling is qualitatively similar at 4 h in sham and CCI mice

Serially sectioned colon tissue from 3 d CCI mice shows that **(A)** HP-1 labeling follows a comparable luminal-to-basal pattern as **(B)** HIF1α labeling supporting the use of HIF1α as a surrogate marker for colon hypoxia. Representative HIF1α labeling from two different **(C)** 4 h sham mice and **(D)** 4 h CCI mice. Qualitatively similar staining characteristics along the depth of the crypts between sham and injured mice suggest that there is no overt difference in the colon’s hypoxic gradient at 4 h after CCI.

|  | 4h | | 8 h | | 1 d | | 3 d | | 1 wk | | 2 wk | | | 4 wk | |  |
| --- | --- | --- | --- | --- | --- | --- | --- | --- | --- | --- | --- | --- | --- | --- | --- | --- |
|  | Sham | CCI | Sham | CCI | Sham | CCI | Sham | CCI | Sham | CCI | Sham | CCI | Sham | | CCI | |
| Intestinal permeability and histology | 2 (1) | 7 (1) | 2 | 8 | 2 | 8 | 3 (1) | 6 (3) |  |  |  |  |  | |  | |
| Microbiome analysis and histology |  |  |  |  |  |  |  |  |  |  |  |  | 6 | | 7 | |
| Histological assessment only | 1 | 3 | 1 |  | 1 |  |  |  | 4 | 7 | 4 | 8 |  | |  | |
| **Total mice used for histology** | **3** | **10** | **3** | **8** | **3** | **8** | **3** | **6** | **4** | **7** | **4** | **8** | **6** | | **7** | |
| Hypoxia assessment |  |  |  |  | 4 | 6 | 7 | 7 |  |  |  |  |  | |  | |
| **Total mice per timepoint** | **4** | **11** | **3** | **8** | **7** | **14** | **11** | **16** | **4** | **7** | **4** | **8** | **6** | | **7** | |

**Supplemental Table 1: Allocation of mice by assay, timepoint and injury condition**

Mice utilized for the intestinal permeability assessment were also utilized for histological assessments (crypt depth, crypt-villi distance, and goblet cell assessment). Mice utilized for microbiome analysis were also used for histological assessments at 4 wks. Additional mice were used solely for histological assessment at unique timepoints (1 wk, 2 wk) or to increase group sizes. Mice generated for colon hypoxia assessment were not included in any other analysis. Numbers in parentheses represent mice excluded from analysis as detailed in the main text.

|  | **Pre-Sham** | **1d Sham** | **2d Sham** | **3d Sham** | **1wk Sham** | **2wk Sham** | **4wk Sham** | **Pre-CCI** | **1d CCI** | **2d CCI** | **3d CCI** | **1wk CCI** | **2wk CCI** | | **4wk CCI** |  | |
| --- | --- | --- | --- | --- | --- | --- | --- | --- | --- | --- | --- | --- | --- | --- | --- | --- | --- |
| **Pre-Sham** | x | 0.730 | 0.660 | 0.123 | 0.281 | 0.284 | 0.828 | 0.015 | 0.176 | 0.341 | 0.096 | 0.020 | 0.060 | | 0.001 | *p*-values | |
| **1d Sham** | 0.851 | x | 0.759 | 0.362 | 0.914 | 0.657 | 0.728 | 0.036 | 0.264 | 0.223 | 0.311 | 0.136 | 0.132 | | 0.002 |  |  |
| **2d Sham** | 0.851 | 0.851 | x | 0.768 | 0.537 | 0.510 | 0.791 | 0.002 | 0.012 | 0.104 | 0.011 | 0.043 | 0.044 | | 0.001 |  |  |
| **3d Sham** | 0.553 | 0.689 | 0.851 | x | 0.777 | 0.763 | 0.637 | 0.008 | 0.037 | 0.060 | 0.024 | 0.190 | 0.205 | | 0.001 |  |  |
| **1wk Sham** | 0.562 | 0.923 | 0.851 | 0.851 | x | 0.722 | 0.760 | 0.046 | 0.183 | 0.157 | 0.183 | 0.440 | 0.361 | | 0.002 |  |  |
| **2wk Sham** | 0.562 | 0.851 | 0.851 | 0.851 | 0.851 | x | 0.601 | 0.070 | 0.170 | 0.267 | 0.324 | 0.423 | 0.247 | | 0.039 |  |  |
| **4wk Sham** | 0.872 | 0.851 | 0.851 | 0.851 | 0.851 | 0.851 | x | 0.035 | 0.272 | 0.566 | 0.127 | 0.134 | 0.286 | | 0.014 |  |  |
| **Pre-CCI** | 0.170 |  |  |  |  |  |  | x | 0.746 | 0.260 | 0.123 | 0.245 | 0.149 | | 0.098 |  |  |
| **1d CCI** |  | 0.562 |  |  |  |  |  | 0.851 | x | 0.884 | 0.490 | 0.208 | 0.272 | | 0.024 |  |  |
| **2d CCI** |  |  | 0.553 |  |  |  |  | 0.562 | 0.911 | x | 0.692 | 0.194 | 0.230 | | 0.003 |  |  |
| **3d CCI** |  |  |  | 0.170 |  |  |  | 0.553 | 0.851 | 0.851 | x | 0.166 | 0.022 | | 0.002 |  |  |
| **1wk CCI** |  |  |  |  | 0.807 |  |  | 0.562 | 0.562 | 0.562 | 0.562 | x | 0.741 | | 0.149 |  |  |
| **2wk CCI** |  |  |  |  |  | 0.562 |  | 0.562 | 0.562 | 0.562 | 0.170 | 0.851 | x | | 0.185 |  |  |
| **4wk CCI** |  |  |  |  |  |  | 0.170 | 0.553 | 0.170 | 0.074 | 0.074 | 0.562 | 0.562 | | x |  | |
|  | *q*-values | | | | | | | | | | | | |  | | |  |

Supplemental Table 2: Significance matrix of unadjusted and FDR-adjusted p-values from post-hoc pairwise comparisons of Bray-Curtis Dissimilarity

Each cell represents a comparison between the corresponding row and column titles. Row and column titles are matched to show comparison of each timepoint and injury condition compared against all others. An “x” has been placed at matched group comparisons. Values above the diagonal line represent the unadjusted p-value, and values below the diagonal line represent *p*-values from comparisons of interest that are adjusted for multiple testing (*q*-values) by Benjamini, Krieger, and Yekutieli two-stage linear step-up procedure (Q:1%). Empty cells represent comparisons that were not meaningful and, as such, were not included in multiple testing correction (n_sham_ = 6, n_CCI_ = 7).

|  | **Pre-Sham** | **1d Sham** | **2d Sham** | **3d Sham** | **1wk Sham** | **2wk Sham** | **4wk Sham** | **Pre-CCI** | **1d CCI** | **2d CCI** | **3d CCI** | **1wk CCI** | **2wk CCI** | **4wk CCI** | |  | |
| --- | --- | --- | --- | --- | --- | --- | --- | --- | --- | --- | --- | --- | --- | --- | --- | --- | --- |
| **Pre-Sham** | x | 0.544 | 0.281 | 0.059 | 0.220 | 0.412 | 0.953 | 0.034 | 0.387 | 0.471 | 0.126 | 0.045 | 0.081 | 0.002 | | *p* - values | |
| **1d Sham** | 0.690 | x | 0.382 | 0.136 | 0.925 | 0.774 | 0.616 | 0.048 | 0.410 | 0.471 | 0.592 | 0.199 | 0.093 | 0.001 | |  |  |
| **2d Sham** | 0.570 | 0.603 | x | 0.390 | 0.367 | 0.498 | 0.497 | 0.007 | 0.043 | 0.228 | 0.066 | 0.104 | 0.041 | 0.003 | |  |  |
| **3d Sham** | 0.324 | 0.518 | 0.603 | x | 0.675 | 0.755 | 0.288 | 0.007 | 0.122 | 0.136 | 0.035 | 0.443 | 0.151 | 0.002 | |  |  |
| **1wk Sham** | 0.544 | 0.954 | 0.603 | 0.777 | x | 0.652 | 0.448 | 0.095 | 0.339 | 0.355 | 0.156 | 0.639 | 0.318 | 0.013 | |  |  |
| **2wk Sham** | 0.603 | 0.833 | 0.649 | 0.830 | 0.768 | x | 0.414 | 0.181 | 0.444 | 0.614 | 0.456 | 0.745 | 0.314 | 0.059 | |  |  |
| **4wk Sham** | 0.963 | 0.762 | 0.649 | 0.570 | 0.616 | 0.603 | x | 0.094 | 0.492 | 0.755 | 0.120 | 0.159 | 0.431 | 0.033 | |  |  |
| **Pre-CCI** | 0.247 |  |  |  |  |  |  | x | 0.729 | 0.162 | 0.025 | 0.185 | 0.132 | 0.048 | |  |  |
| **1d CCI** |  | 0.603 |  |  |  |  |  | 0.820 | x | 0.815 | 0.179 | 0.236 | 0.445 | 0.109 | |  |  |
| **2d CCI** |  |  | 0.544 |  |  |  |  | 0.520 | 0.858 | x | 0.387 | 0.269 | 0.242 | 0.003 | |  |  |
| **3d CCI** |  |  |  | 0.247 |  |  |  | 0.247 | 0.520 | 0.603 | x | 0.251 | 0.027 | 0.002 | |  |  |
| **1wk CCI** |  |  |  |  | 0.768 |  |  | 0.520 | 0.544 | 0.570 | 0.520 | x | 0.413 | 0.116 | |  |  |
| **2wk CCI** |  |  |  |  |  | 0.598 |  | 0.518 | 0.616 | 0.544 | 0.247 | 0.603 | x | 0.189 | |  |  |
| **4wk CCI** |  |  |  |  |  |  | 0.247 | 0.297 | 0.518 | 0.074 | 0.074 | 0.518 | 0.520 | x | |  | |
|  | *q* - values | | | | | | | | | | | | | |  | |  |

Supplemental Table 3: Significance matrix of unadjusted and FDR-adjusted p-values from post-hoc pairwise comparisons of weighted UniFrac distance

Each cell represents a comparison between the corresponding row and column titles. Row and column titles are matched to show comparison of each timepoint and injury condition compared against all others. An “x” has been placed at matched group comparisons. Values above the diagonal line represent the unadjusted p-value, and values below the diagonal line represent *p*-values from comparisons of interest that are adjusted for multiple testing (*q*-values) by Benjamini, Krieger, and Yekutieli two-stage linear step-up procedure (Q:1%). Empty cells represent comparisons that were not meaningful and, as such, were not included in multiple testing correction (n_sham_ = 6, n_CCI_ = 7).
